# Supplementary material for: Analysis of 3760 hematologic malignancies reveals rare transcriptomic aberrations of driver genes
Source: Genome Med. 2024 May 20;16:70. doi: 10.1186/s13073-024-01331-6 (PMC11103968; doi:10.1186/s13073-024-01331-6)
Supplement: Supplementary file 1 — Additional file 1: Supplementary Figures, Supplementary Material and Methods, Supplementary Results. PDF file containing the accompanying supplementary text as well as all Supplementary Figures S1-S31. [file 13073_2024_1331_MOESM1_ESM.pdf]

Analysis of 3,760 hematologic malignancies reveals rare transcriptomic aberrations of driver genes

–

Supplementary Information

Supplementary Materials and Methods

DNA sequencing, read mapping, variant calling, and copy number variations calling

Whole genome sequencing (WGS) libraries were prepared from 1µg of DNA, which was fragmented by ultrasonic shearing (Covaris, Woburn, MA) to a target insert size of 350bp, with the TruSeq PCR free library prep kit following the manufacturer's recommendations (Illumina, San Diego, CA, USA) and 2x150bp paired-end sequences were generated on a NovaSeq 6000 or HiSeqX instrument with 100x coverage (Illumina, San Diego, CA, USA). Reads were aligned to the human reference genome (GRCh37, Ensembl annotation) using the Isaac aligner (v3.16.02.19) (1) through BaseSpace's WGS app (v5, Illumina, San Diego, CA, USA) with default parameters. Resulting BAM files were used in BaseSpace's Tumor/Normal app (v3) to call single nucleotide variants and short insertions and deletions (<50bp) with Strelka (v2.4.7) (2) and large-scale structural variants with Manta (v0.28.0) (3). As no sample-specific normal tissue was available, a so-called unmatched normal was used in its place to reduce technical artifacts and

germline calls. For this, WGS was performed on sex-matched genomic DNA from a mixture of multiple anonymous donors (Promega, Fitchburg, WI, USA). Copy number variations were called with GATK CallCopyRatioSegments (v4.0.8.1) (4) using the Broad Institute's recommended best practices CNV pipeline. Here, two panels of normals (PON) were used for denoising, consisting of 124 female and 191 male samples, which presented a normal karyotype during routine diagnostics.

RNA sequencing, read mapping, and fusion detection

TruSeq Total Stranded RNA kit was used, starting with 250 ng of total RNA, to generate RNA libraries following the manufacturer's recommendations (Illumina, San Diego, CA, USA). 2×100 bp paired-end reads were sequenced on the NovaSeq 6000 (Illumina, San Diego, CA, USA) with a median of 50 million reads per sample. Using BaseSpace's RNA-seq Alignment app (v2.0.1) with default parameters, reads were mapped with STAR aligner (v2.5.0a) (5) to the human reference genome hg19 (RefSeq annotation). Single nucleotide variants and short insertions and deletions were called using Isaac variant caller (v2.3.13)(1). Fusion calling was performed with Manta (v0.29.0)(3), Arriba (v1.2.0) (6), and STAR-Fusion (v1.9.0) (7). Only fusions that were called by at least two algorithms were considered in order to reduce false positives.

NB-act

NB-act (Negative Binomial activation) estimates the probability of a gene that is generally not expressed in the population to be expressed in a particular sample. It was applied to the rarely-expressed genes of all samples from the dataset.

We assumed that the observed count k_{ij} of gene $i = 1,\dots,p$ in sample $j = 1,\dots,m$ followed a negative binomial distribution with a shared dispersion parameter θ and expected value μ_{ij}:

$$P(k_{ij}) = NB(k_{ij}|\mu_{ij}, \theta)$$

The dispersion parameter θ was set to 0.02 and corresponds to the empirically observed lowest dispersion values estimated by OUTRIDER on expressed genes. As dispersion inversely relates to variance, this value makes NB-act conservative. OUTRIDER uses 1 fragment per kilobase of transcript per million mapped reads (FPKM) as the upper expression limit for a lowly-expressed gene. Therefore, we set the expected count μ_{ij} of a gene is given as the product of 1 FPKM, the sample-specific size factor s_j, and the exon length of the gene L_i [nt] divided by 1,000 to get absolute count expectations:

$$\mu_{ij} := s_j L_i / 1000$$

The size factors s_j capture variations in sequencing depth and were estimated using DESeq2 (8). The exon length L_i [nt] was extracted from Gencode v33b. The *P*-values were calculated and corrected for multiple testing using functions provided by OUTRIDER (‘alternative=greater’). All significant outliers of this analysis were named “activation outliers”.

<u>CGC</u>

The Cancer Gene Census (CGC) is a catalog of those genes that contain mutations that have been causally implicated in cancer and explains how the dysfunction of these genes drives cancer (9). The CGC genes GRCh37 v97 were obtained from https://cancer.sanger.ac.uk/census and subsetted for the subsequent analysis, including enrichment, association, and machine learning model (Table S18).

Table S18. CGC subsetting criteria

Subset	Column ‘Tissue Type’	Column ‘Role in Cancer’	Number of genes
CGC cancer driver gene	any	any	721

CGC cancer oncogene	any	containing ‘oncogene’	314
CGC cancer tumor suppressor gene	any	containing ‘TSG’	318
CGC hematologic malignancy driver gene	containing ‘L’*	any	322
CGC hematologic oncogene	containing ‘L’*	containing ‘oncogene’	156
CGC hematologic tumor suppressor gene	containing ‘L’*	containing ‘TSG’	134

*L: leukemia or lymphoma

The cancer type of the gene was determined by the ‘Tissue Type’ annotation. All genes were labeled as ‘cancer’. Genes whose ‘Tissue Type’ included ‘L’ (leukemia or lymphoma) were further labeled as ‘leukemia’. The role of the gene was determined by the ‘Role in Cancer’ annotation. Genes whose ‘Role in Cancer’ included ‘oncogene’ or ‘TSG’ were labeled as ‘oncogene’ or ‘tumor suppressor gene’ respectively. Some genes have been reported with both roles and can have received both labels.

Hematologic panel genes

The gene panel consists of genes that are required for diagnosis and have been found to be associated with prognosis or are relevant for therapy in hematologic malignancies (10,11). The gene panel furthermore contains genes that have been described in the literature as being recurrently mutated in one or more hematologic disease entities. Altogether, these were 122

genes, partially overlapping CGC hematologic malignancy driver genes. These 122 genes were used for curation, sample genotyping analysis, and machine learning models:

APC, ARID1A, ASXL1, ASXL2, ATM, ATRX, BCL2, BCOR, BCORL1, BIRC3, BRAF, BRCC3, BTK, CALR, CARD11, CBL, CCND1, CD79A, CD79B, CDH23, CDKN2A, CEBPA, CHEK2, CREBBP, CSF3R, CSNK1A1, CTCF, CUX1, CXCR4, DDX3X, DDX41, DDX54, DHX29, DIS3, DNMT3A, EP300, ETNK1, ETV6, EZH2, FAM46C, FANCL, FAS, FAT4, FBXW10, FBXW7, FLT3, FOXO1, GATA1, GATA2, GNAS, GNB1, GPR98, ID3, IDH1, IDH2, IKBKB, IL2RG, JAK1, JAK2, JAK3, KDM5A, KDM6A, KIT, KLF2, KLHL6, KMT2D, KRAS, LRP1B, MAP2K1, MAPK1, MEF2B, MPL, MYBBP1A, MYC, MYD88, NF1, NFKBIE, NOTCH1, NOTCH2, NPM1, NRAS, PHF6, PIGA, PLCG2, POT1, PPM1D, PRPF8, PTPN11, PTPRD, RAD21, RB1, RPS15, RUNX1, SETBP1, SF1, SF3A1, SF3B1, SH2B3, SMC1A, SMC3, SRSF2, STAG2, STAT3, STAT5B, SUZ12, TBL1XR1, TCF3, TET2, TLR2, TNFAIP3, TNFRSF14, TP53, TRAF3, U2AF1, U2AF2, UBR5, WHSC1, WT1, XPO1, ZBTB7A, ZMYM3, ZRSR2

Driver gene prediction

Features

For each gene, the result of each tool was summarized as numerical values, which reflected its mutation, expression, and splicing aberrations profile. Therefore, all genes were assigned with vectors of numbers, resulting in a feature matrix (genes x features). The feature vectors were generated for each of the tools in two ways:

1) Per study group: using only samples from each of the 14 study groups
2) Complete dataset: using all samples

OUTRIDER features

For each study group and for the complete dataset, the 22 OUTRIDER features per gene were generated, which consisted of:

1) The number of samples for which the gene is among the top 1, 5, 10, 25, and 50 overexpression outliers sorted by False Discovery Rate (FDR) on the one hand and underexpression outlier on the other hand (10 features)
2) The number of samples for which the gene is among the significant overexpression outliers for FDR cutoffs of 0.01, 0.05, and 0.1 on the one hand and among underexpression outliers on the other hand (6 features)
3) The number of samples for which the gene z-score is larger than 2, 4, or 6 on the one hand and less than -2, -4, -6 on the other hand (6 features)

NB-act features

For each study group and for the complete dataset, the 11 NB-act features per gene were generated, which consisted of:

1) The number of samples for which the gene is among the top 1, 5, 10, 25, and 50 overexpression outliers sorted by FDR (5 features)
2) The number of samples for which the gene is among the significant overexpression outliers for FDR cutoffs of 0.01, 0.05, 0.1 (3 features)
3) The number of samples for which the gene z-score is larger than 2, 4, or 6 (3 features)

FRASER features

For each study group and for the complete dataset, the 22 FRASER features per gene were generated, which consisted of:

1) The number of samples for which the gene is among the top 1, 5, 10, 25, and 50 overrepresented splicing outliers sorted by FDR on the one hand, and underrepresented splicing outliers on the other hand (10 features)
2) The number of samples for which the gene is among the significant overrepresented splicing outliers for FDR cutoffs of 0.01, 0.05, and 0.1 on the one hand and among underrepresented splicing outliers on the other hand (6 features)
3) The number of samples for which the gene delta Intron Jaccard Index is larger than 0.1, 0.2, 0.3 on the one hand, and less than -0.1, -0.2, -0.3 on the other hand (6 features)

AbSplice features

For each study group and for the complete dataset, the 9 AbSplice features were generated per gene, which consisted of:

1) The maximum AbSplice-DNA score across all rare variants and samples by cutoffs 0.01, 0.05, 0.2 (3 features)
2) The mean AbSplice-DNA score across all rare variants and samples by cutoffs 0.01, 0.05, 0.2 (3 features)
3) The number of samples for which the gene has at least one rare variant with an AbSplice-DNA score larger than 0.01, 0.05, 0.2 (3 features)

IntOGen features

The IntOGen feature vectors were generated separately from the result of the seven individual methods. The evaluation metrics of the seven methods were chosen the same way as IntOGen and then transformed accordingly (Table S19). The mode of action (e.g., role as an oncogene or tumor suppressor gene) was calculated the same way as IntOGen. Each transformed metric was assigned into the corresponding category ‘Activating’, ‘Loss-of-function’, or ‘ambiguous’ based on the mode of action, resulting in 7*3=21 features.

Table S19. Seven methods and the corresponding metric, filter, and transformation

Method	Metric and transformation	Filter
HotMAPS (12)	-log(q-value)	-
OncodriveCLUSTL (13)	sum(SCORE)	-
smRegions (14)	max(U)	-
OncodriveFML (15)	-log(Q_VALUE)	-
MutPanning (16)	-log(FDR)	Significance < 0.1
dNdScv (17)	-log(qallsubs_cv)	-
CBaSE (18)	-log(q_pos)	-

Gene functional features

Co-essential modules (19) were obtained from https://static-content.springer.com/esm/art%3A10.1038%2Fs41588-021-00840-z/MediaObjects/41588_2021_840_MOESM4_ESM.zip and transformed into a gene times module matrix, with 0 indicating the absence of the gene in the module and 1 denoting its presence. Embedding Omics and embedding STRING (20) were retrieved from https://academic.oup.com/nargab/article-lookup/doi/10.1093/nargab/lqad095#supplementary-data Table S3 and S5.

Random forest classifier

The random forests were trained using the function ‘RandomForestClassifier’ with ‘n_estimators’ = 100, ‘criterion’ = ‘gini’, ‘max_depth’ = 10, ‘min_samples_split’ = 19, ‘min_samples_leaf’ = 1, ‘min_weight_fraction_leaf’ = 0, ‘max_features’ = ‘auto’, ‘max_leaf_nodes’ = None, ‘min_impurity_decrease’ = 0, ‘bootstrap’ = True, ‘oob_score’ = False’, ‘random_state’ = None, ‘verbose’ = 0, ‘warm_start’ = False‘, class_weight’ = None, ‘ccp_alpha’ = 0, and ‘max_samples’ = None.

Logistic regression

Logistic regression was trained using the function ‘LogisticRegression’ (Python package scikit-learn v1.0.2 (21)) with the solver set to ’lbfgs’, the penalty set to ‘none’, the maximum number of iterations to 100,000, and default settings otherwise.

XGBoost

XGBoost was trained using the function ‘XGBClassifier’ (Python package xgboost v1.6.1 (22)) with the tree method set to ‘hist’. Parameter search was performed using ‘RandomizedSearchCV’ (Python package scikit-learn v1.0.2 (21)) with the number of parameter settings that are sampled set to 50. The parameter search searched for L1 and L2 regularization terms on weights (xgb’s alpha and xgb’s lambda) between 0.01 to 10,000 using the function ‘loguniform’ (Python package spicy v1.8.0 (23)). Default settings were applied elsewhere.

Artificial neural network

A multilayer perceptron with two hidden layers of size 512 was trained using Adam optimizer (‘weight_decay’ = 1-5). The model was trained for 400 epochs with a learning rate of 0.0001, optimizing the binary cross-entropy loss. We used the Python package Pytorch 2.1.0 (24).

Supplementary Results

Comparative analysis of matched vs. unmatched variant calling and filtering approaches

To assess the effects of not having matched controls on variant calling, we performed a comparative analysis leveraging 57 matched germline normal samples at 100x coverage for 57 tumor samples (48 AML, 5 CML, 3 CLL, and 1 MCL). We created variant calls for these 57 samples using the same pipeline employed in our study but using the sample-specific normal control tissue in place of the anonymous unmatched normals.

For cancer with low purity, rare variants with a within-sample frequency close to 50% can be suspected to be germline variants. However, in our data across 57 samples for which matched control samples were available, the distribution of VAF indicated high tumor purity (Figure S31), which is in agreement with a previous report on leukemia samples of the TCGA dataset (25). Consequently, we did not apply a high-frequency VAF cutoff.

This matched-normal variant set was then compared to the unmatched-normal variants in order to assess the degree of overlap for different filtering regimes. Notably, we observed in our calls a peak in the distribution around VAF 10% both when using matched and unmatched controls, which is absent from the TCGA-LAML WGS data (Figure S31), suggesting an excess of low-frequency variants in our data that are more likely false calls. These false calls may originate from the Strelka variant calling pipeline, which was chosen at the start of this project (2). Another non-exclusive explanation is that we are fragmenting DNA using an acoustic shearing procedure known to lead to false variant calls in this frequency range (26) and may not all be excluded by our bioinformatics pipeline. Therefore, we decided to filter out variants with a VAF of less than 10% or less than 15%.

Altogether, the following call sets were used:

Matched-normal:

i) (matched_filter1) QUALITY==’PASS’.

ii) (matched_filter2) QUALITY==’PASS’; keep VAF >= 0.10

Unmatched-normal:

iii) (unmatched_filter1) QUALITY==’PASS’

iv) (unmatched_filter2) QUALITY==’PASS’; discard gnomAD MAF >= 0.0005; keep VAF >= 0.10

v) (unmatched_filter3) QUALITY==’PASS’; discard gnomAD MAF >= 0.0005; keep VAF >= 0.15; sequencing depth>=20

We summarize these results in the tables below by reporting mean±std over samples and computing the following metrics:

Precision

=true_positives/(false_positives+true_positives)

=matched∩unmatched/(unmatched_only+matched∩unmatched)

Recall

= true_positives/(false_negatives+true_positives)

=matched∩unmatched/(matched_only+matched∩unmatched).

Comparison between:

- (matched_filter1) QUALITY==’PASS’
- (unmatched_filter1) QUALITY==’PASS’

The overlap over 57 samples

**matched_filter1∩unmatched_filter1**	4,986±5,009
**matched_filter1 only**	4,770±4,225
**unmatched_filter1 only**	432,070±169,409

The averaging recall, precision, and f1-score over 57 samples

**precision**	0.011±0.010
**recall**	0.510±0.093
**f1-score**	0.022±0.018

Comparison between:

- (matched_filter1) QUALITY==’PASS’
- (unmatched_filter2) QUALITY==’PASS’; discard MAF >= 0.0005; keep VAF >= 0.10;

The overlap over 57 samples

**matched_filter1∩unmatched_filter2**	3,093±2,935
**matched_filter1 only**	6,663±6,922
**unmatched_filter2 only**	58,144±22,909

The averaging recall, precision, and f1-score over 57 samples

**precision**	0.049±0.033

**recall**	0.355±0.129
**f1-score**	0.082±0.047

Comparison between:

- (matched_filter1) QUALITY=='PASS'
- (unmatched_filter3) QUALITY=='PASS'; discard MAF >= 0.000,5; keep VAF >= 0.15; sequencing depth>=20

The overlap over 57 samples

**matched_filter1∩unmatched_filter3**	2,086±1,484
**matched_filter1 only**	7,670±8,188
**unmatched_filter3 only**	43,047±18,802

The averaging recall, precision, and f1-score over 57 samples

**precision**	0.048±0.029
**recall**	0.282±0.141
**f1-score**	0.076±0.040

Compared to unmatched_filter1, unmatched_filter2 led to better precision but worse recall for matched calls. The filtering also significantly improved the f1-score (paired t-test, *P*-value < $6x10^{-20}$). Compared with unmatched_filter1, unmatched_filter3 also led to better precision but worse

recall. Yet unmatched_filter3 showed slightly lower precision and recall compared to unmatched_filter2.

Given that matched_filter1 contains a large peak of low-frequency variants suspected to be artifacts, we repeated parts of the analysis using matched_filter2 as a reference point, where we excluded all variants with VAF<0.1 from the matched normal calls. The results are provided in the tables below.

Comparison between:

- (matched_filter2) QUALITY=='PASS'; keep VAF >= 0.10;
- (unmatched_filter2) QUALITY=='PASS'; discard MAF >= 0.000,5; keep VAF >= 0.10;

The overlap over 57 samples

**matched_filter2∩unmatched_filter2**	3,093±2,935
**matched_filter2 only**	2,112±2,130
**unmatched_filter2 only**	58,144±22,909

The averaging recall, precision, and f1-score over 57 samples

**precision**	0.049±0.033
**recall**	0.598±0.083
**f1-score**	0.088±0.052

Comparison between:

- (matched_filter2) QUALITY=='PASS'; keep VAF >= 0.10;
- (unmatched_filter3) QUALITY=='PASS'; discard MAF >= 0.000,5; keep VAF >= 0.15; sequencing depth>=20

The overlap over 57 samples

**matched_filter2∩unmatched_filter3**	2,086±1,484
**matched_filter2 only**	3,120±3,656
**unmatched_filter3 only**	43,047±18,802

The averaging recall, precision, and f1-score over 57 samples

**precision**	0.048±0.029
**recall**	0.456±0.139
**f1-score**	0.083±0.043

Remarkably, the exclusion of low VAF variants from the matched normal calls, which were already filtered out from the unmatched normal calls, did not impact precision but resulted in a doubling of recall. This suggests using unmatched_filter2 for our further analysis (Figure 3 and Figures S4 - S8, S15, S16) as it retained the majority (at least ~60%) of somatic variants. These analyses indicate that our filtered variants capture the majority of somatic variants, but these are a minority of our filtered variants. The majority of filtered variants are likely rare germline variants.

Interestingly, "unmatched_filter3" showed a better performance than "unmatched_filter2" when used as input for the IntOGen workflow in the leukemia driver gene prediction task (Figure S18). Therefore, we applied this filter for our prediction model (Figures 4 and Figures S19-S27).

Supplementary Figures

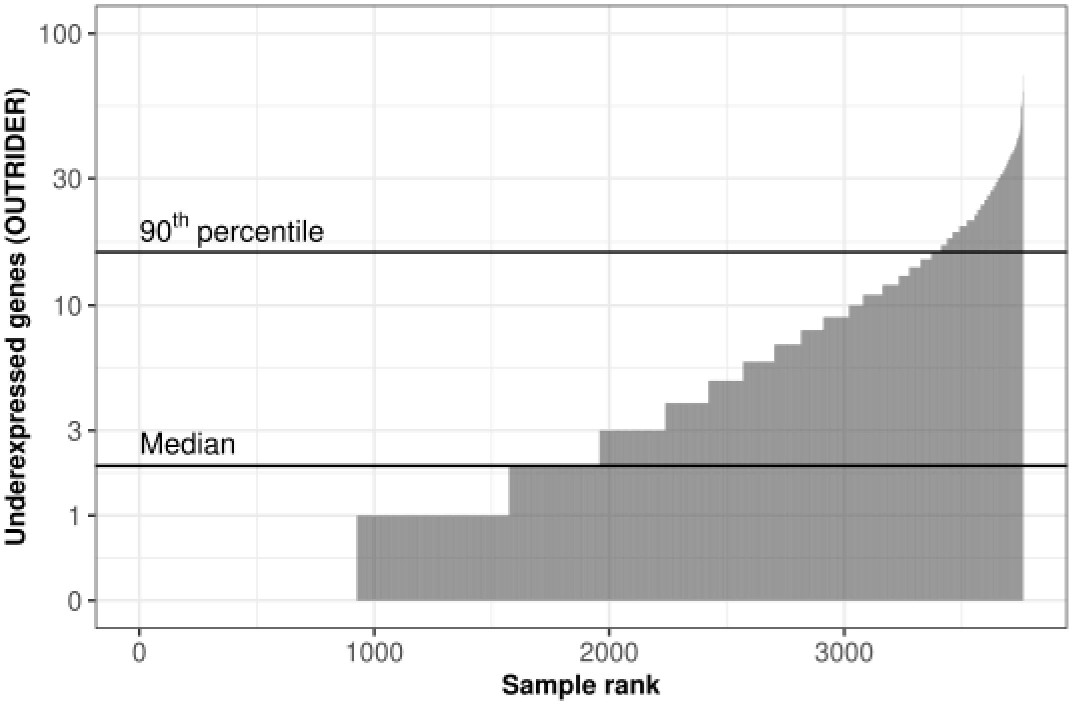

Figure S1. Underexpressed genes (OUTRIDER) per sample. Horizontal lines mark the median and the 90th percentile.

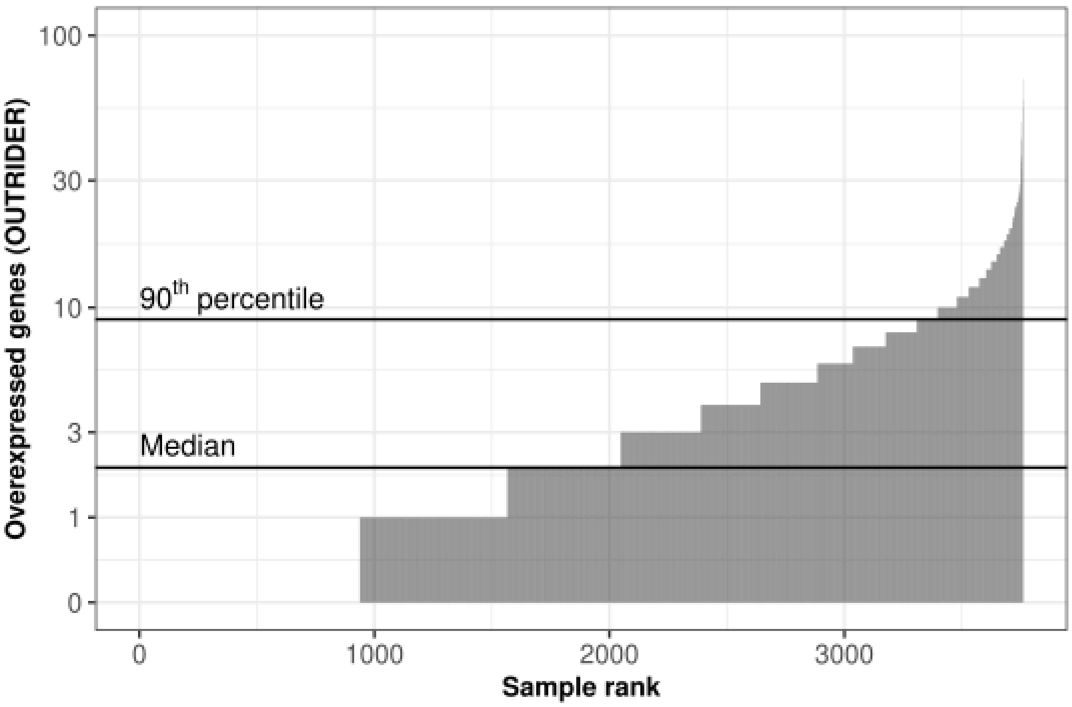

Figure S2. Overexpressed genes (OUTRIDER) per sample. Horizontal lines mark the median and the 90th percentile.

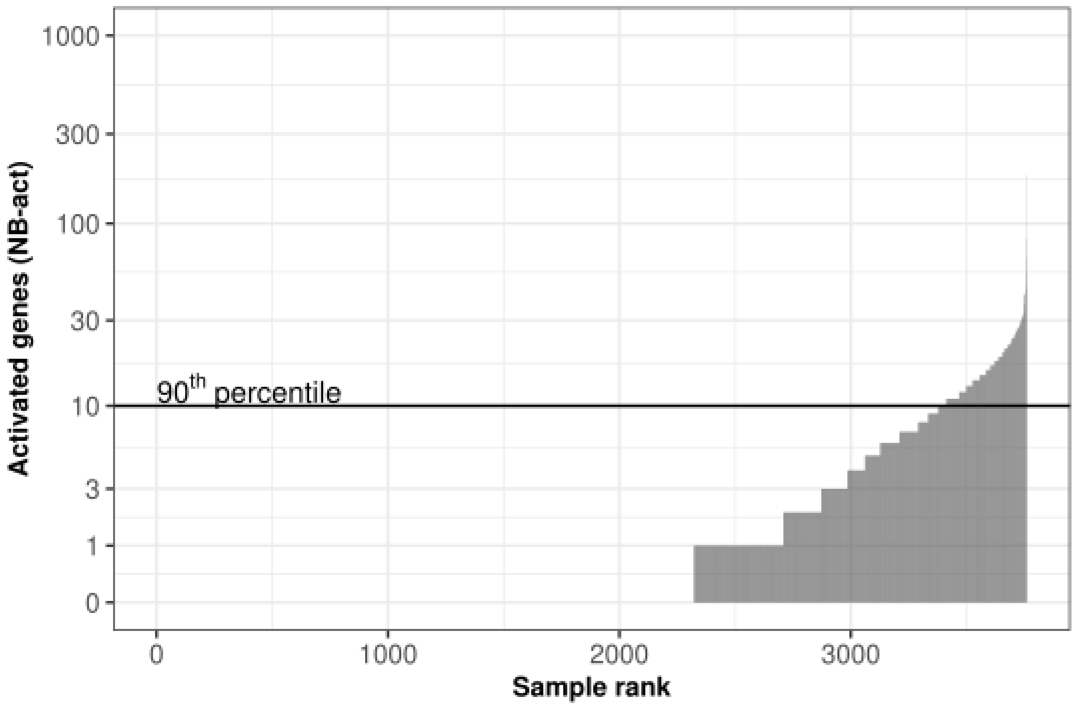

Figure S3. Activated genes (NB-act) per sample. The horizontal line marks the 90th percentile.

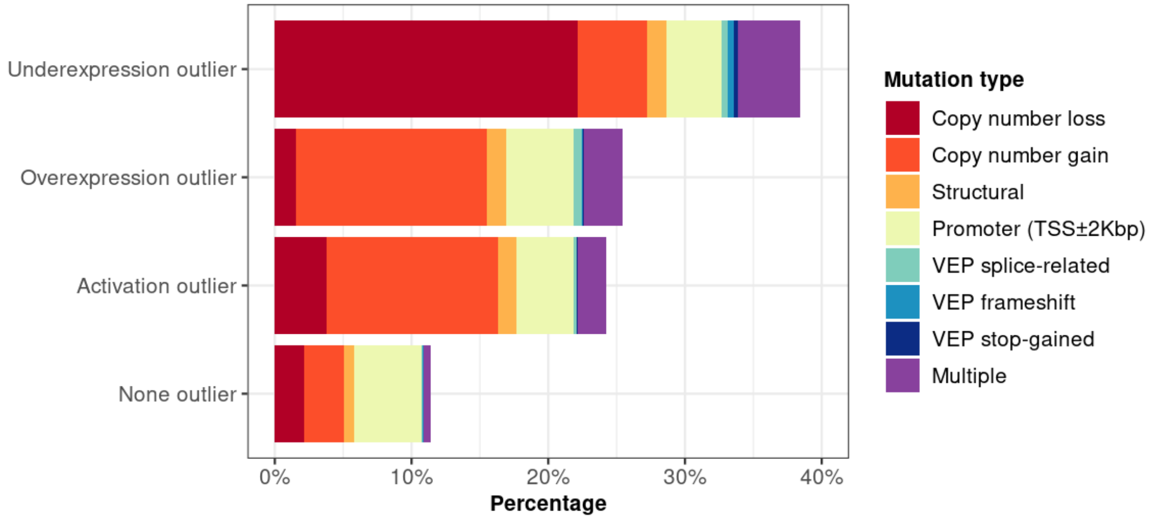

Figure S4. Percentage of gene sample combinations that are or are not detected as expression outliers, colored by their mutation type. Copy number losses and variants likely triggering nonsense-mediated decay (splice-related, frameshift, and stop-gained) are found in the regions of underexpression outliers, while copy number gains are found around overexpression outliers and activation outliers. All categories are exclusive. Instances overlapping multiple categories are categorized as ”Multiple”.

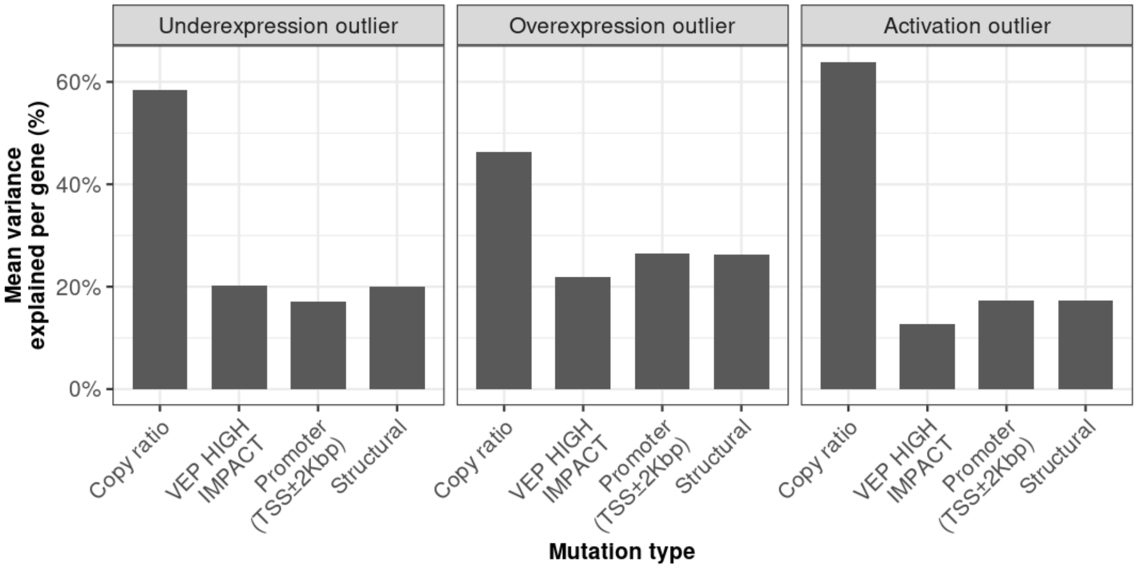

Figure S5. Variance component analysis for gene expression levels, showing the average proportion of variance explained by different mutation types. For this analysis, the categories can overlap. For instance, a gene can harbor both a VEP high-impact variant and an altered copy ratio in a given sample.

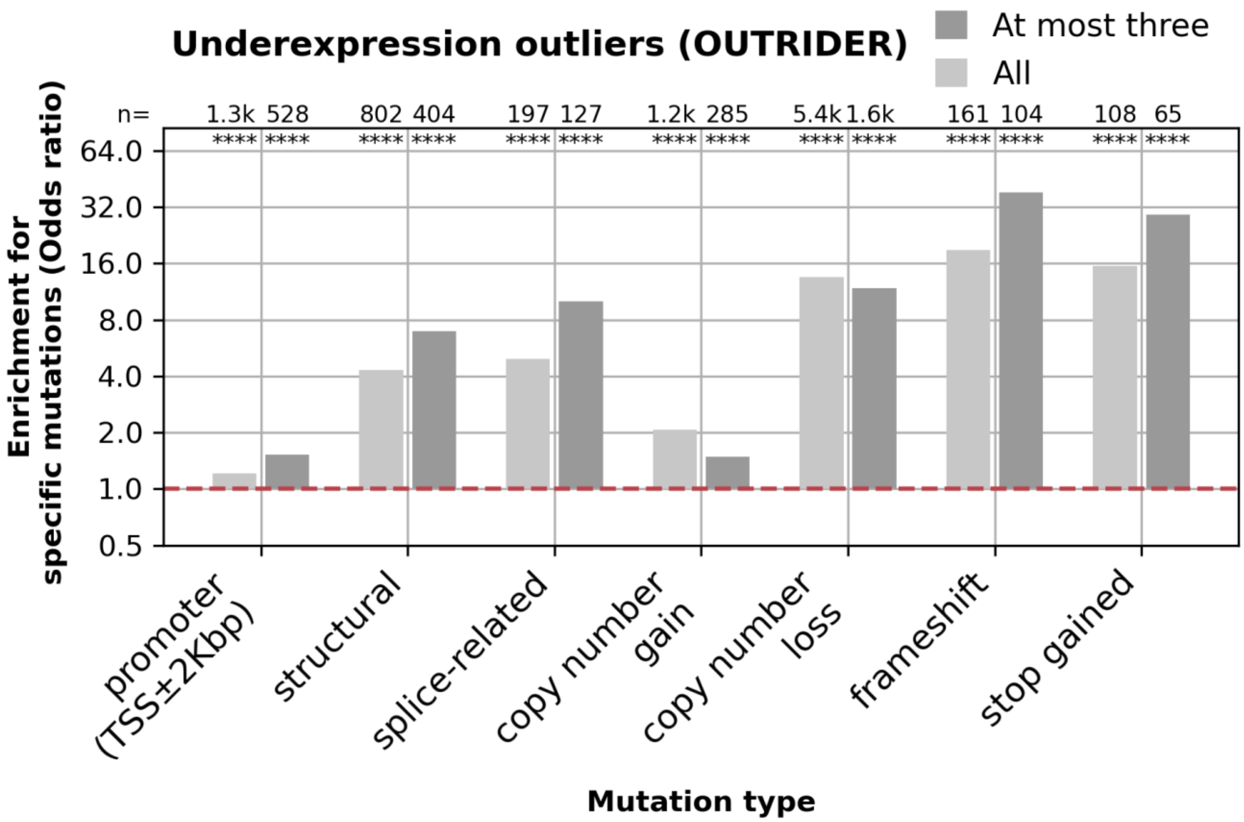

Figure S6. Enrichment for different mutation types among all genes called by OUTRIDER as well as at most three significant genes per sample that were called to be underexpression outliers. Numbers of the mutations and nominal significances from the Fisher test are labeled at the top of the bars. (ns: not significant; *: P ≤ 0.05; **: P ≤ 0.01; ***: P ≤ 0.001; ****: P ≤ 0.0001)

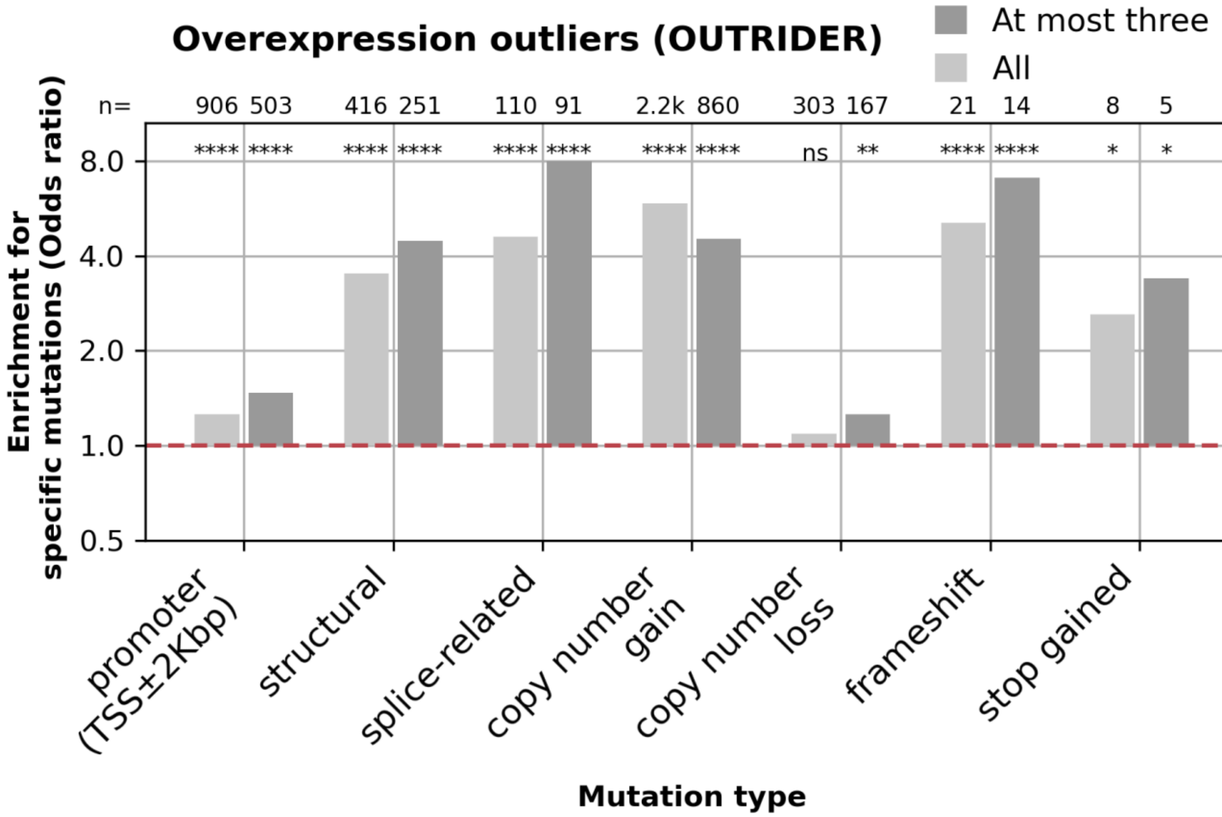

Figure S7. Enrichment for different mutation types among all genes called by OUTRIDER as well as at most three significant genes per sample that were called to be overexpression outliers. Numbers of the mutations and nominal significances from the Fisher test are labeled at the top of the bars. (ns: not significant; *: P ≤ 0.05; **: P ≤ 0.01; ***: P ≤ 0.001; ****: P ≤ 0.0001)

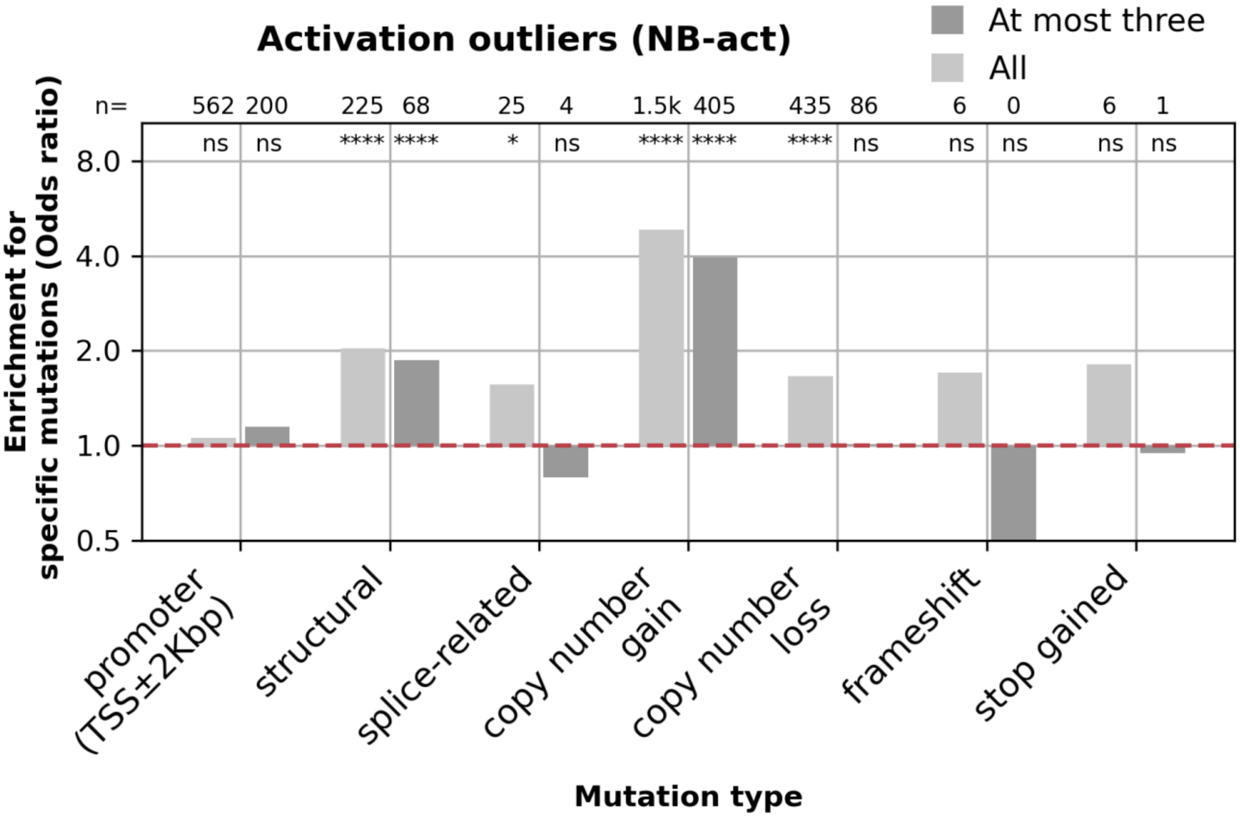

Figure S8. Enrichment for different mutation types among all genes called by NB-act as well as at most three significant genes per sample that were called to be activation outliers. Numbers of the mutations and nominal significances from the Fisher test are labeled at the top of the bars. (ns: not significant; *: P ≤ 0.05; **: P ≤ 0.01; ***: P ≤ 0.001; ****: P ≤ 0.0001)

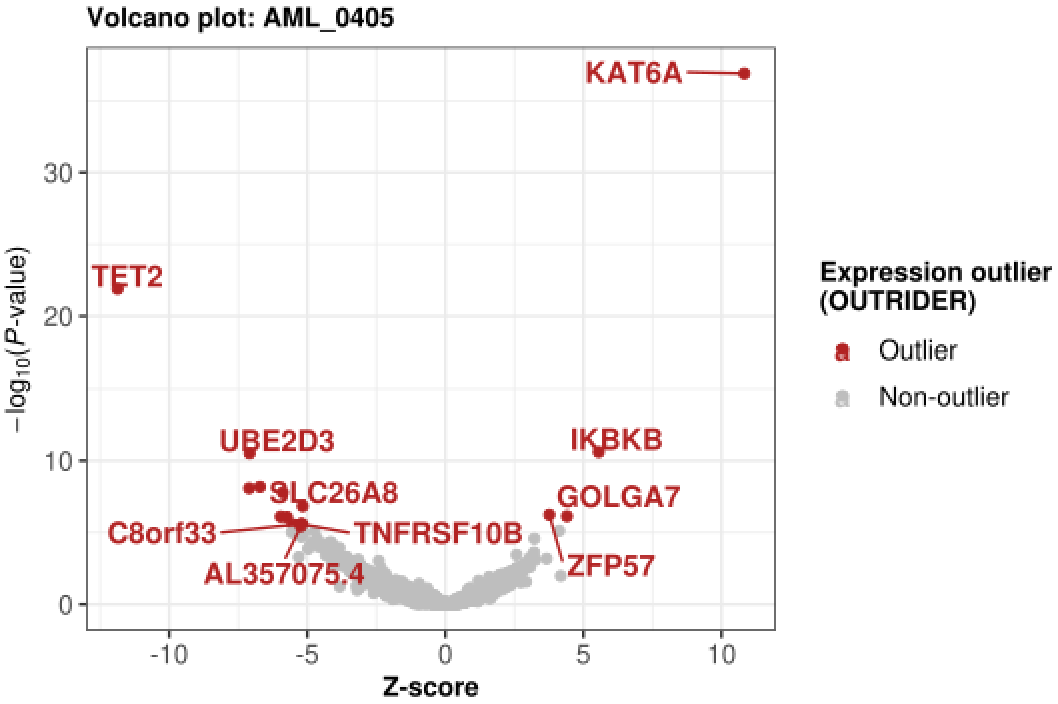

Figure S9. *P*-value against z-score of expression outliers in sample AML_0405. Four overexpression outliers and 13 underexpression outliers (both red) were detected.

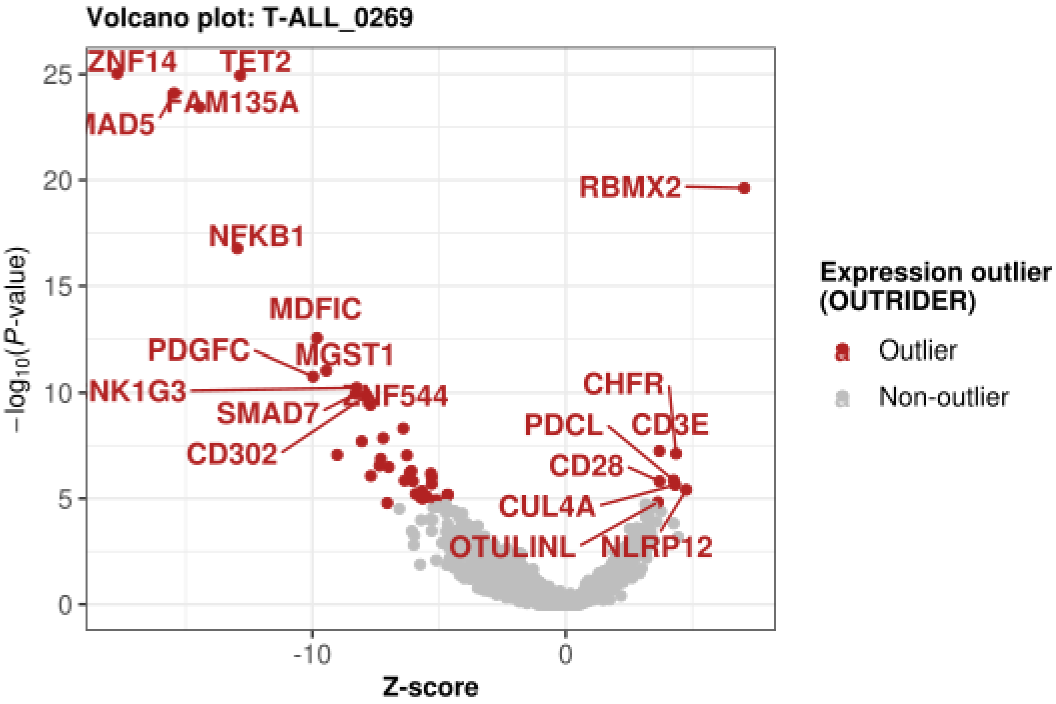

Figure S10. *P*-value and z-score of expression outliers in sample T-ALL_0269. Eight overexpression outliers and 38 underexpression outliers were detected (both red).

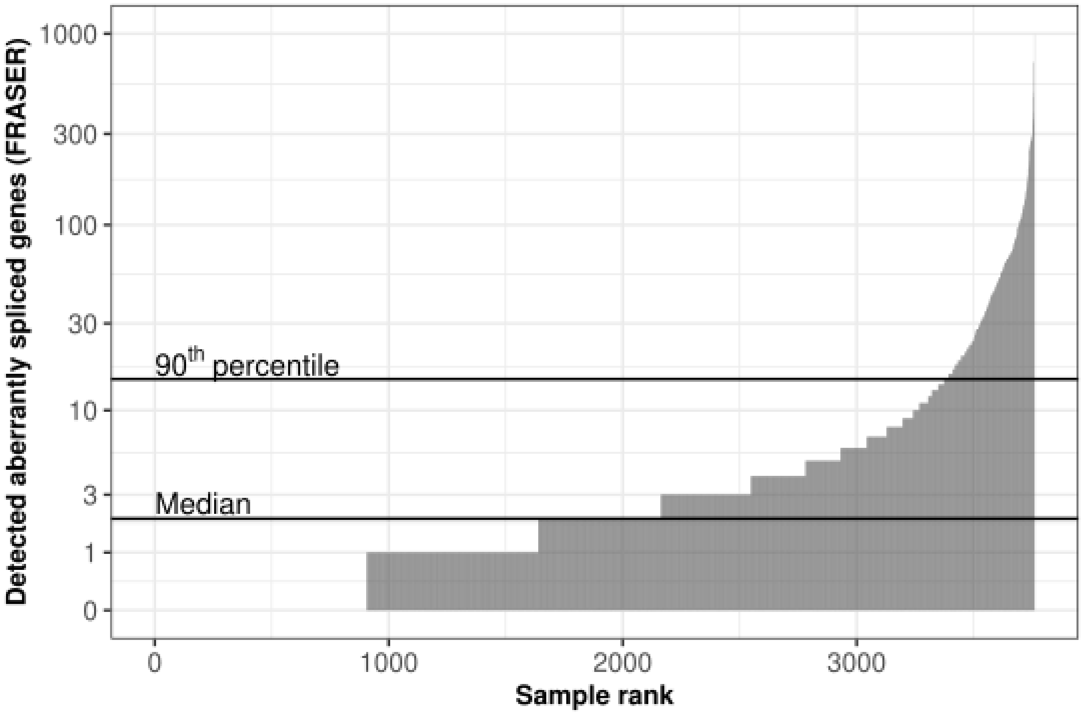

Figure S11. Aberrantly spliced genes (FRASER) per sample. Horizontal lines mark the median and the 90th percentile.

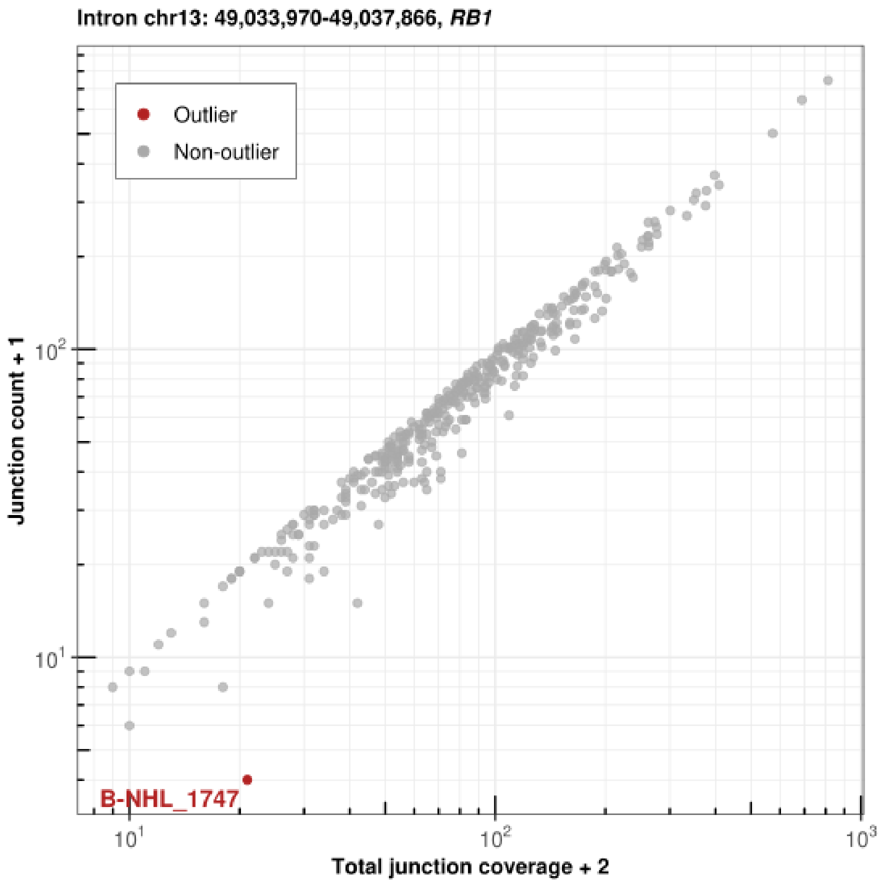

Figure S12. Junction counts (split reads) against the displayed intron's total junction coverage (exon-intron or intron-exon spanning reads) of the *RB1* case study. The displayed intron of the *RB1* only shows significant abnormal splicing in sample B-NHL_1747.

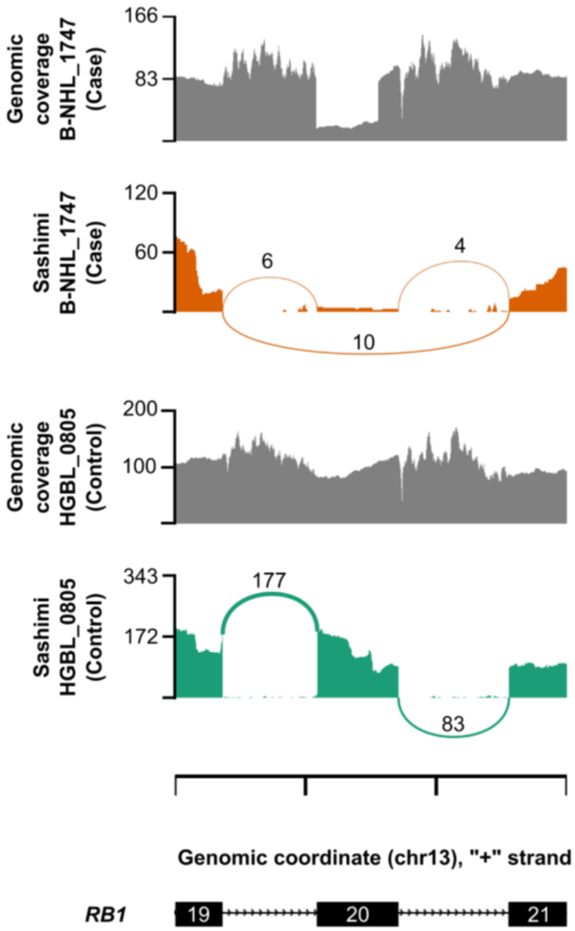

Figure S13. Genomic coverage and sashimi plots of *RB1* case study. Genomic coverage of WGS is shown (y-axis, gray). Sashimi plots showing RNA-seq read coverage (y-axis) and the numbers of split reads spanning an intron indicated on the exon-connecting line for two aberrant splicing events. One case individual (orange) showing exon-skipping for *RB1* 20th and one control individual (green) are displayed. The rare splice-affecting structural variant, which existed exclusively in this case individual, is observed in the genomic coverage plot as a drop of coverage.

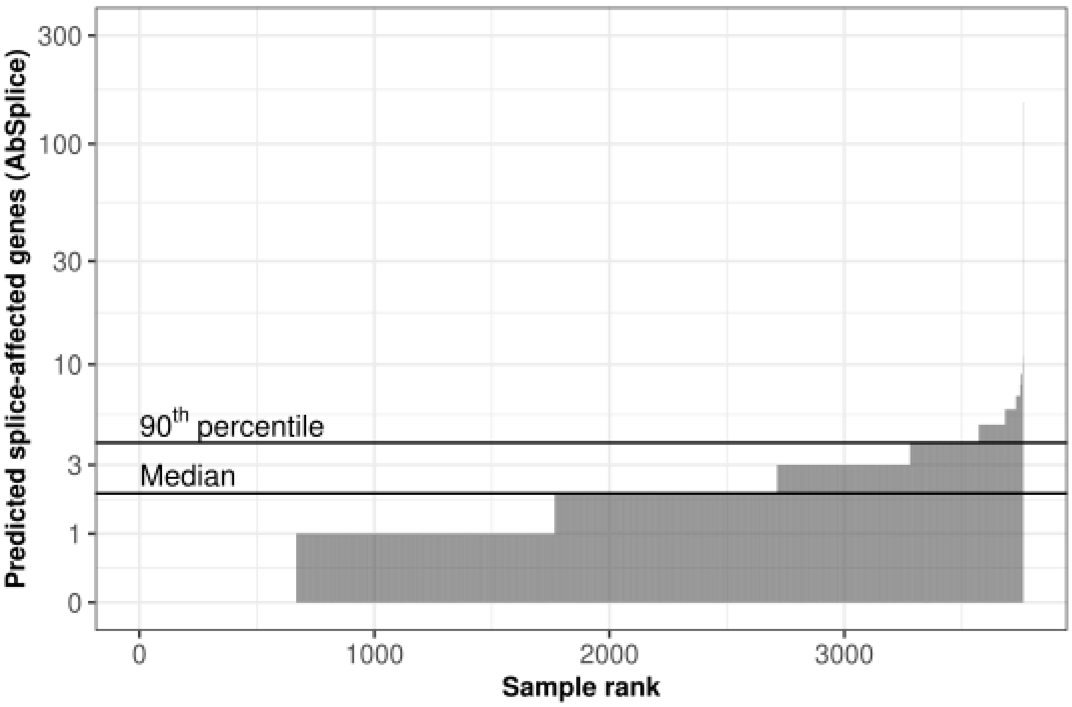

Figure S14. Predicted splice-affected genes (AbSplice) per gene. Horizontal lines mark the median and the 90th percentile.

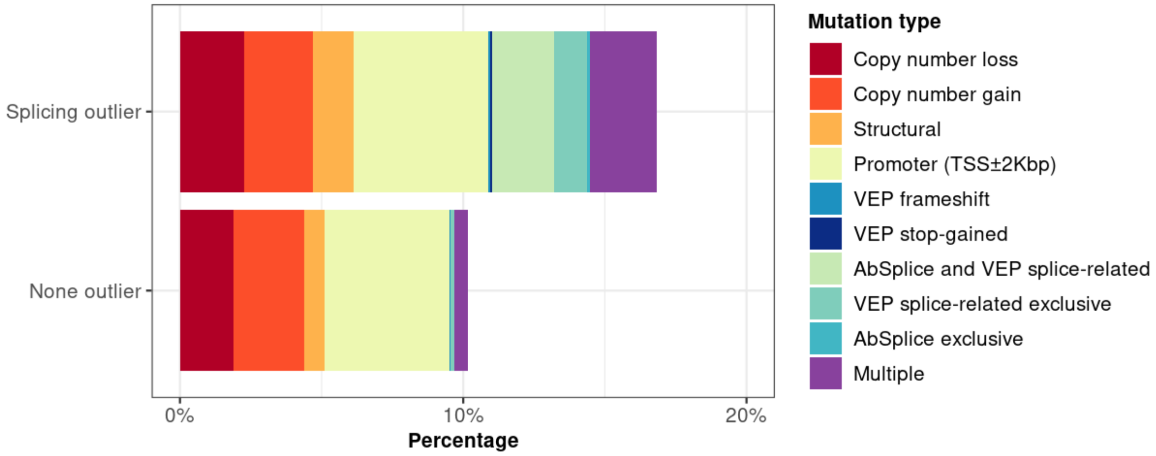

Figure S15. Percentage of gene sample combinations that have or do not have splicing outliers, colored by detected mutation types. The ratio of copy number variations did not change between gene sample combinations being splicing outliers or not, while AbSplice and VEP splice-related variants are strongly enriched in splicing outliers.

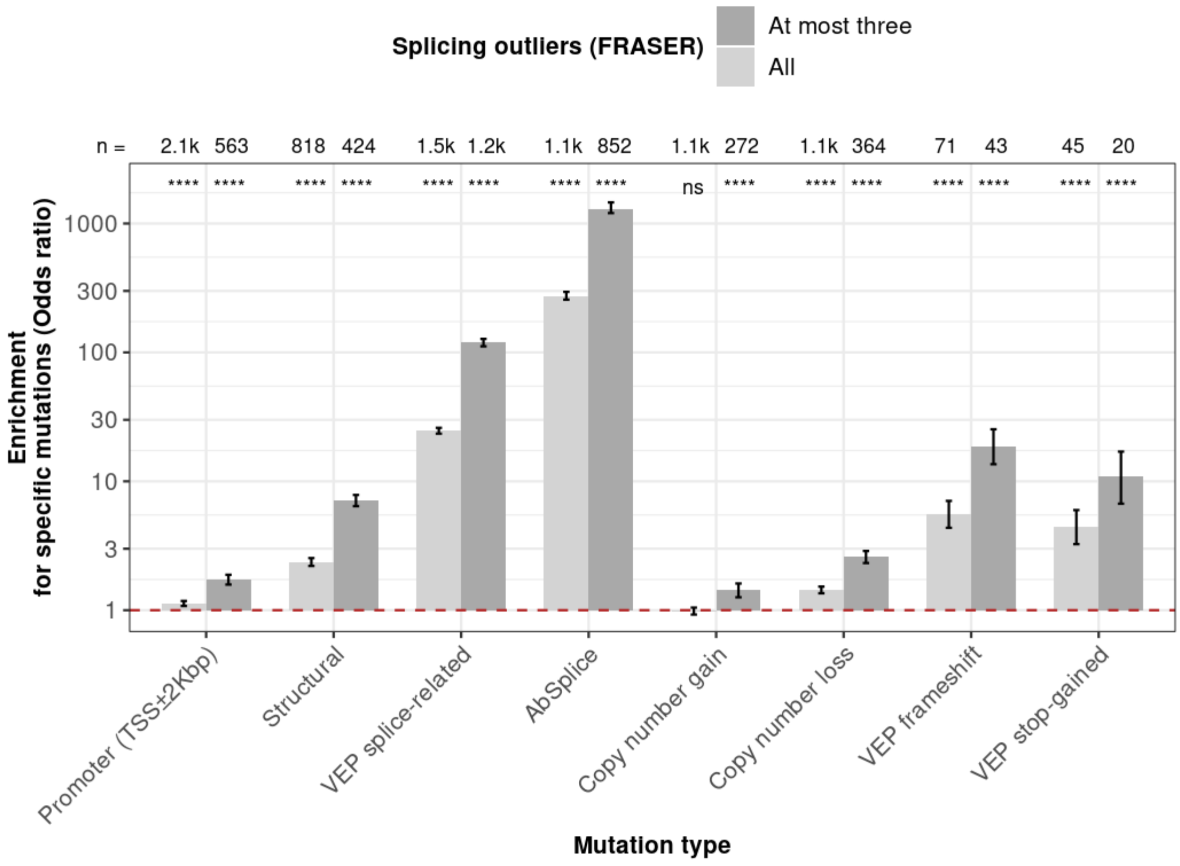

Figure S16. Enrichment for different mutation types among all genes called by FRASER as well as at most three significant genes per sample that were called to be splicing outliers. Numbers of the mutations and nominal significances from the Fisher test are labeled at the top of the bars (ns: not significant; *: P ≤ 0.05; **: P ≤ 0.01; ***: P ≤ 0.001; ****: P ≤ 0.0001).

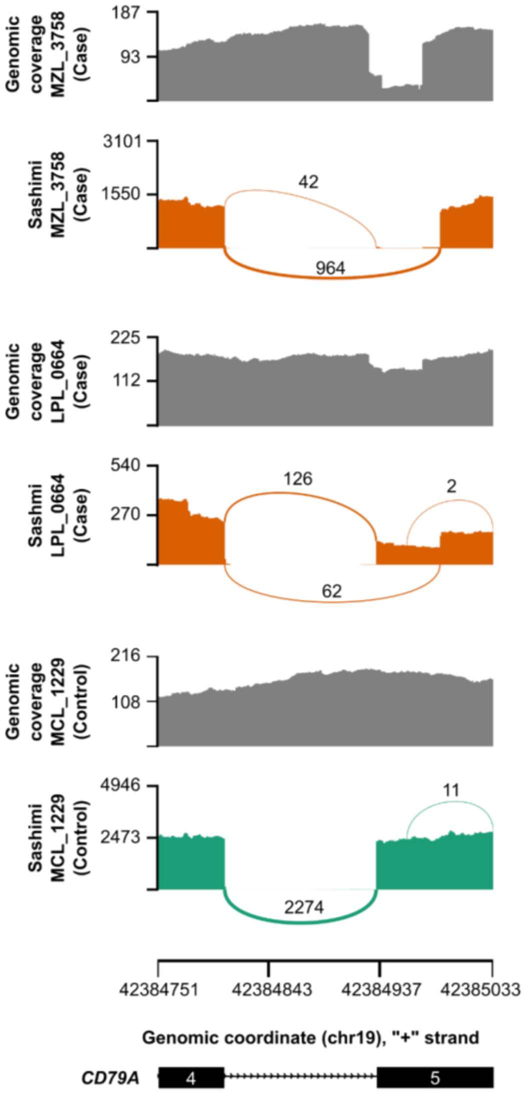

Figure S17. Genomic coverage and sashimi plots of *CD79A* case study. Genomic coverage of WGS is shown (y-axis, gray). Sashimi plots showing RNA-seq read coverage (y-axis) and the numbers of split reads spanning an intron indicated on the exon-connecting line for two aberrant splicing events. Two case individuals (orange) using a unique splice-site in *CD79A* 5th exon acceptor site and one control individual (green) are displayed. The rare splice-affecting deletion (NM_001783.4:c.568-2_610del) predicted by AbSplice, which existed exclusively in this two case individuals, is observed in the genomic coverage plot as a drop of coverage.

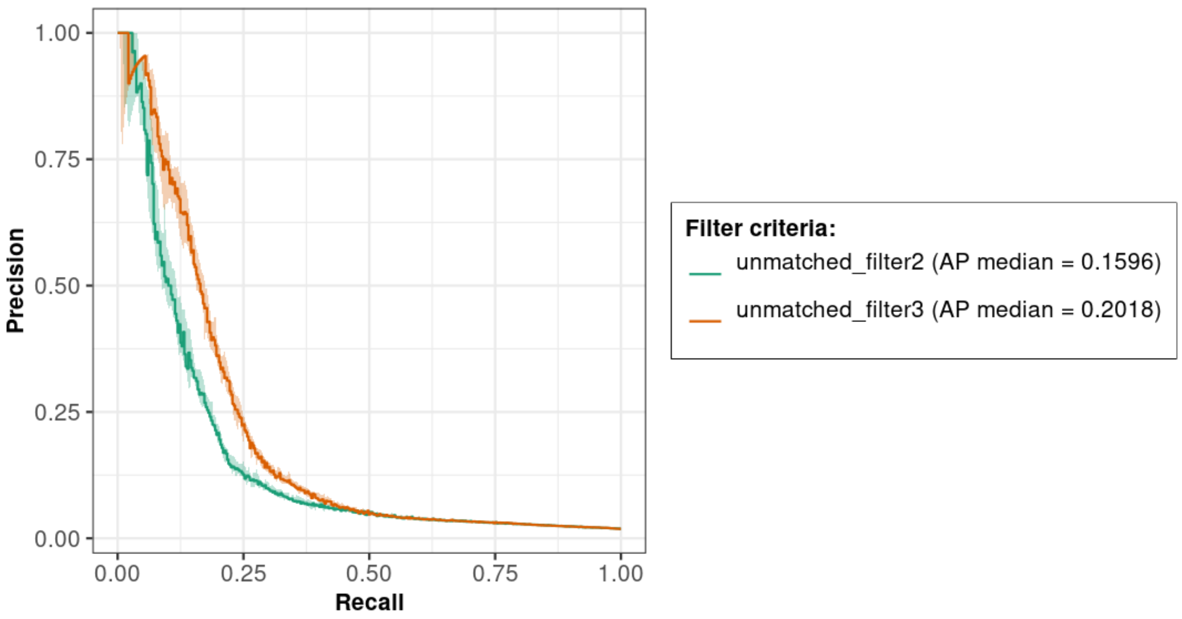

Figure S18. Precision recall curve showing the performance of the hematologic malignancy driver gene prediction model. The input features of the model are the seven IntOGen tools only. Different filter sets were evaluated, as detailed in the Supplementary Results section. Ribbons represent the 80% confidence interval of 10 random repeats.

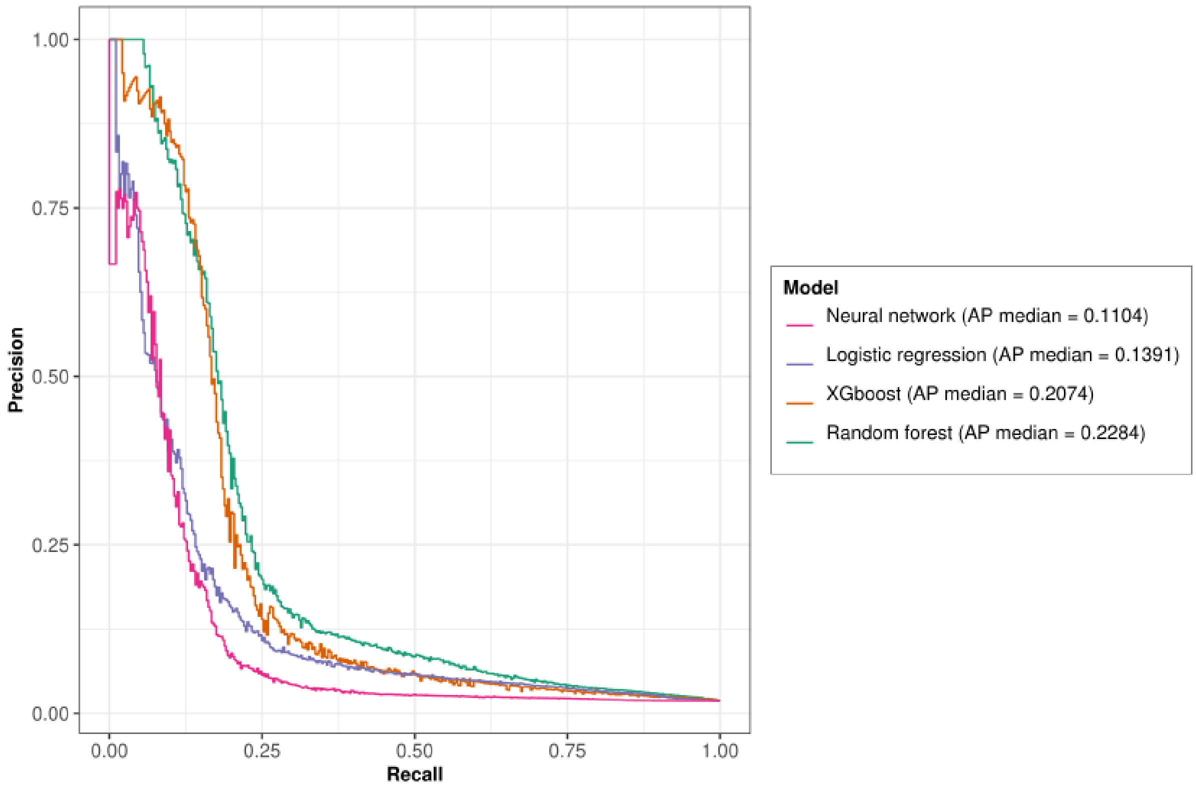

Figure S19. Precision-recall curve showing the performance of the hematologic malignancy driver gene prediction model using different machine learning methods. The median of the average precision (AP) is calculated over 10 random repeats.

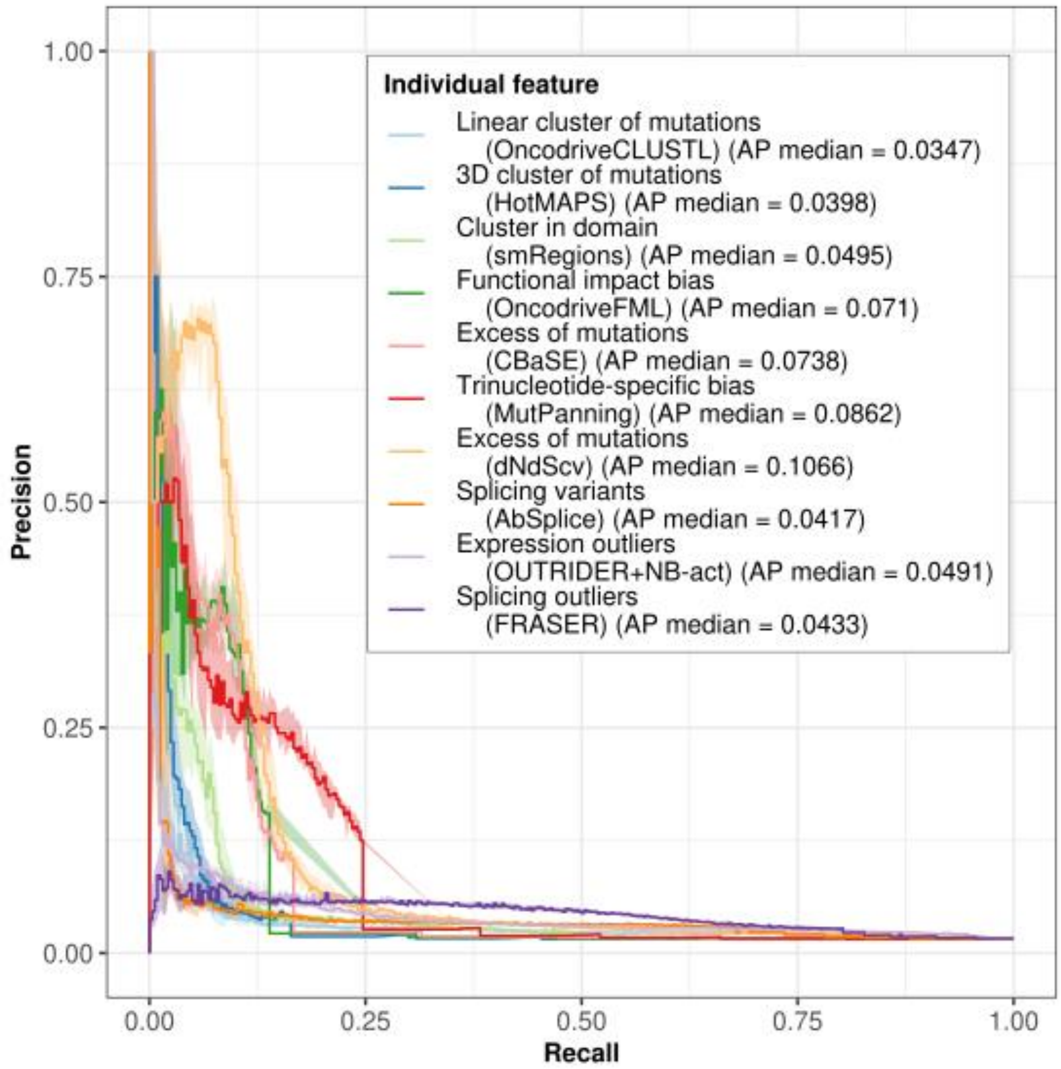

Figure S20. Precision-recall curve showing the performance of the hematologic malignancy driver gene prediction model when using individual features based on the complete dataset. Ribbons represent the 80% confidence interval obtained by 10 random repeats.

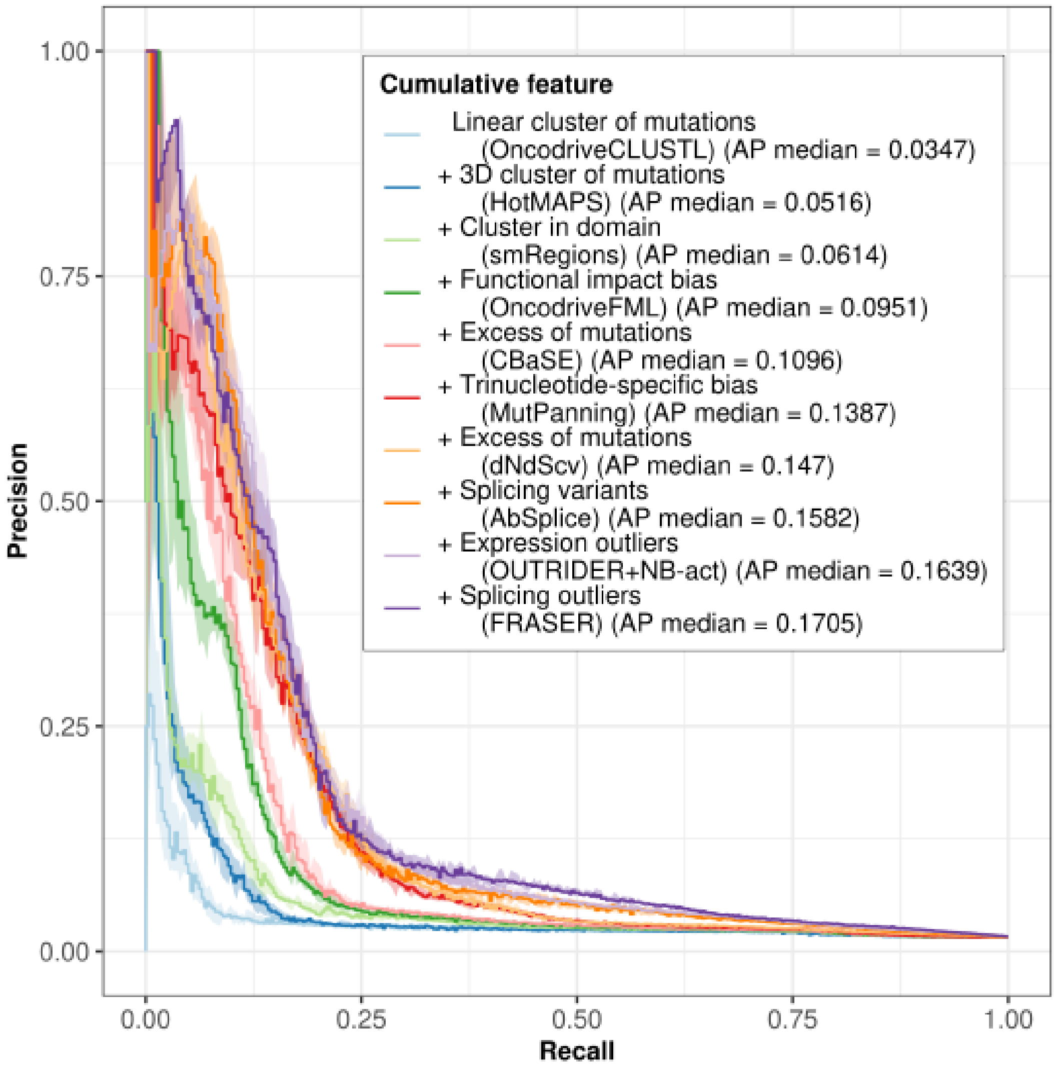

Figure S21. Precision-recall curve showing the performance of the hematologic malignancy driver gene prediction model when adding features cumulatively based on the complete dataset. Ribbons represent the 80% confidence interval obtained by 10 random repeats.

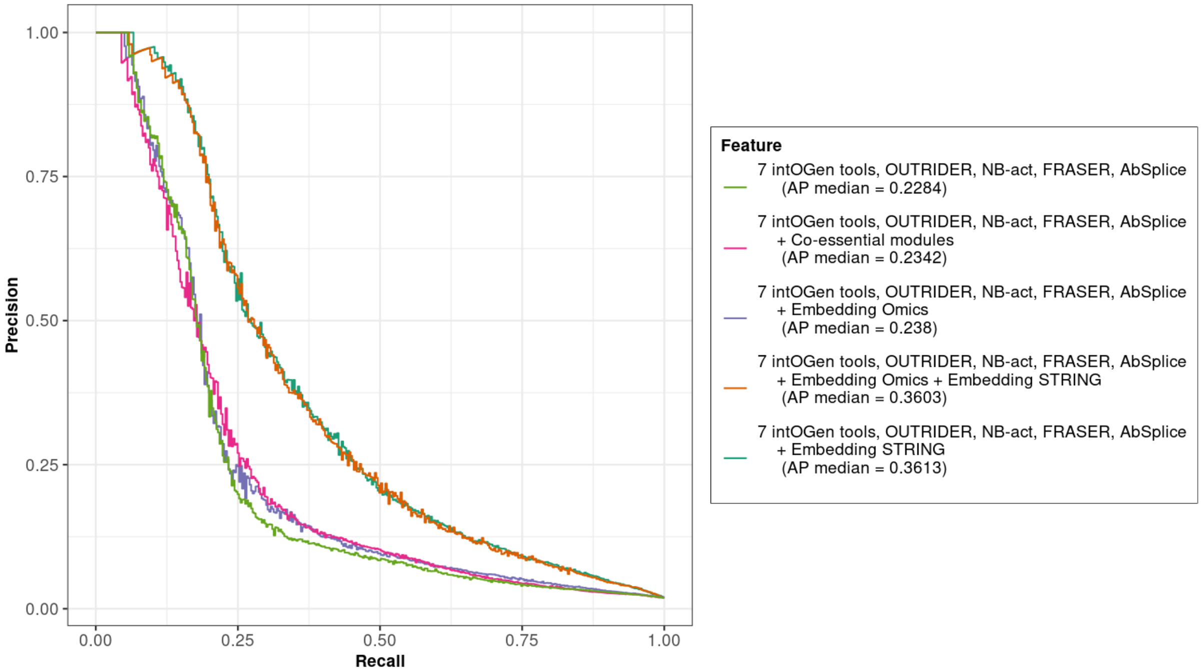

Figure S22. Precision-recall curve showing the performance of the hematologic malignancy driver gene prediction models when adding different external gene functional data as input. The median of average precision (AP) is calculated over 10 random repeats. Performance increases by adding external gene functional data compared to using the seven IntOGen tools, OUTRIDER, NB-act, FRASER, and AbSplice only.

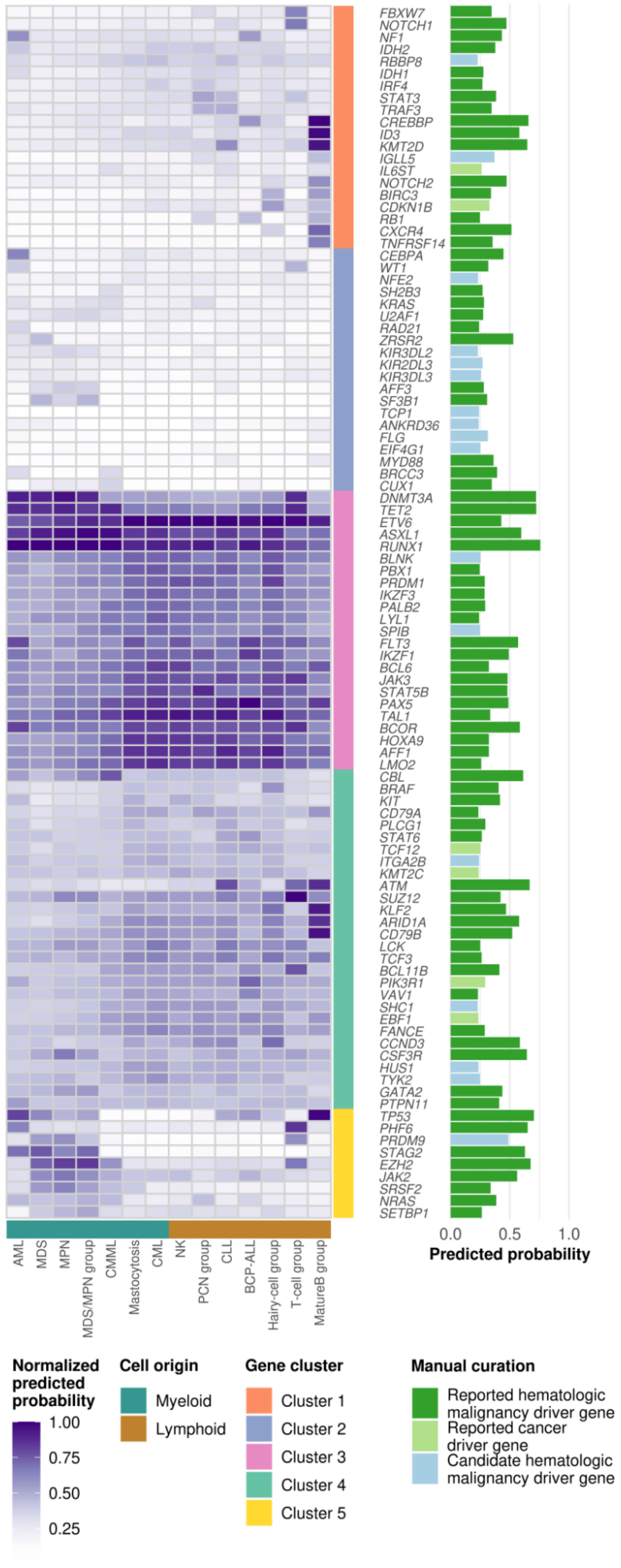
FBXW7
NOTCH1
NF1
IDH2
RBBP8
IDH1
IRF4
STAT3
TRAF3
CREBBP
ID3
KMT2D
IGLL5
IL6ST
NOTCH2
BIRC3
CDKN1B
RB1
CXCR4
TNFRSF14
CEBPA
WT1
NFE2
SH2B3
KRAS
U2AF1
RAD21
ZRSR2
KIR3DL2
KIR2DL3
KIR3DL3
AFF3
SF3B1
TCP1
ANKRD36
FLG
EIF4G1
MYD88
BRCC3
CUX1
DNMT3A
TET2
ETV6
ASXL1
RUNX1
BLNK
PBX1
PRDM1
IKZF3
PALB2
LYL1
SPIB
FLT3
IKZF1
BCL6
JAK3
STAT5B
PAX5
TAL1
BCOR
HOXA9
AFF1
LMO2
CBL
BRAF
KIT
CD79A
PLCG1
STAT6
TCF12
ITGA2B
KMT2C
ATM
SUZ12
KLF2
ARID1A
CD79B
LCK
TCF3
BCL11B
PIK3R1
VAV1
SHC1
EBF1
FANCE
CCND3
CSF3R
HUS1
TYK2
GATA2
PTPN11
TP53
PHF6
PRDM9
STAG2
EZH2
JAK2
SRSF2
NRAS
SETBP1
0.0
0.5
1.0
Predicted probability
AML
MDS
MPN
MDS/MPN group
CMML
Mastocytosis
CML
NK
PCN group
CLL
BCP-ALL
Hairy-cell group
T-cell group
MatureB group
Normalized predicted probability
1.00
0.75
0.50
0.25
Cell origin
Myeloid
Lymphoid
Gene cluster
Cluster 1
Cluster 2
Cluster 3
Cluster 4
Cluster 5
Manual curation
Reported hematologic malignancy driver gene
Reported cancer driver gene
Candidate hematologic malignancy driver gene

Figure S23. Predicted probability from the driver gene prediction model based on the complete dataset and each of the study groups individually, using as features the seven IntOGen tools, OUTRIDER, NB-act, FRASER, AbSplice, and the STRING gene functional embedding. The heatmap shows the predicted driver gene probability per gene (rows) and study group (columns) relative to the column-wise maximum value. The inclusion of gene functional data blurred the differences between disease entities when compared to Figure 4D.

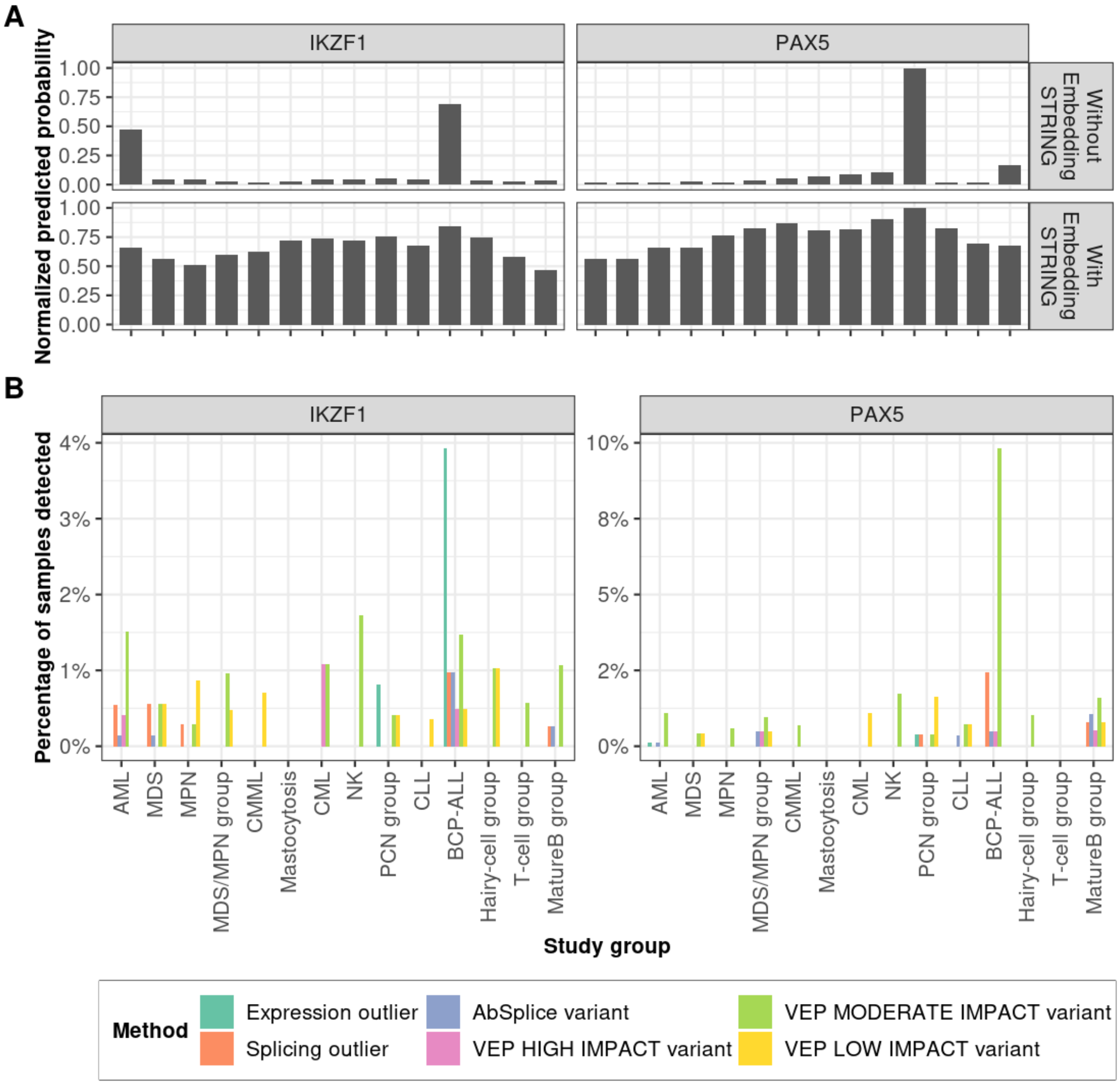

Figure S24. Normalized predicted probabilities and detected aberrations for *IKZF1* and *PAX5*. *IKZF1* and *PAX5* are two genes predicted by the model without external data and the model with external data. A) Normalized predicted probabilities (relative to the disease entity-wise maximum value) using features from the seven IntOGen tools, OUTRIDER, NB-act, FRASER, and AbSplice, with or without Embedding STRING, across each study group. The model using external data predicts a role for those genes in all leukemia entities, whereas the model based on our dataset alone predicts a role for specific entities. B) Percentage of samples detected with corresponding aberrations using VEP, expression outliers, splicing outliers, and AbSplice variants

across each study group. *IKFZ1* and *PAX5* exhibited a high prediction score in BCP-ALL when utilizing leukemia evidence but displayed elevated scores in every study group upon the addition of embeddings. Little or no genomic and transcriptomic aberrations were identified in these genes within other study groups. In this context, the heightened prediction of *IKFZ1* and *PAX5* in other study groups arose exclusively from the external data. This suggests that *IKFZ1* and *PAX5* may function as a driver gene for BCP-ALL but not necessarily for all types of hematologic malignancies.

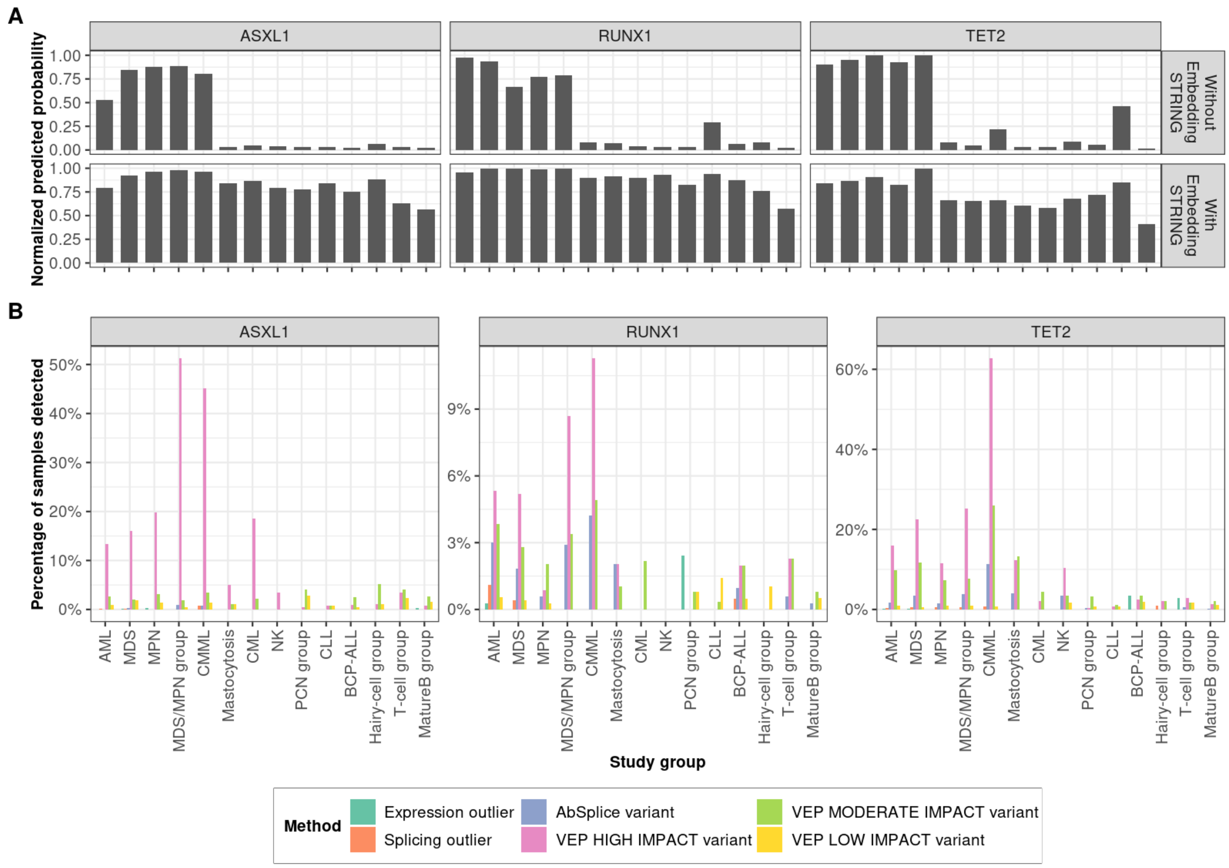

Figure S25. Normalized predicted probabilities and detected aberrations for *ASLX1*, *RUNX1*, and *TET2*. *ASLX1*, *RUNX1*, and *TET2* are two genes predicted by the model without external data and the model with external data. A) Normalized predicted probabilities (relative to the disease entity-wise maximum value) using features from the seven IntOGen tools, OUTRIDER, NB-act, FRASER, and AbSplice, with or without Embedding STRING, across each study group. The model using external data predicts a role for those in all leukemia entities, whereas the model based on our dataset alone predicts a role for specific entities. B) Percentage of samples detected with corresponding aberrations in using VEP, expression outliers, splicing outliers, and AbSplice variants across each study group. *ASLX1, RUNX1,* and *TET2* exhibited a high prediction score in myeloid entities when utilizing leukemia evidence but displayed elevated scores in every study group upon the addition of embeddings. Little or no genomic and transcriptomic aberrations were identified in these genes within other study groups. This aligns

with the known fact that *ASLX1, RUNX1,* and *TET2* function as a driver gene for myeloid entities but not necessarily for all types of hematologic malignancies. In this context, the heightened prediction of *ASLX1, RUNX1,* and *TET2* in other study groups arose exclusively from the embedding.

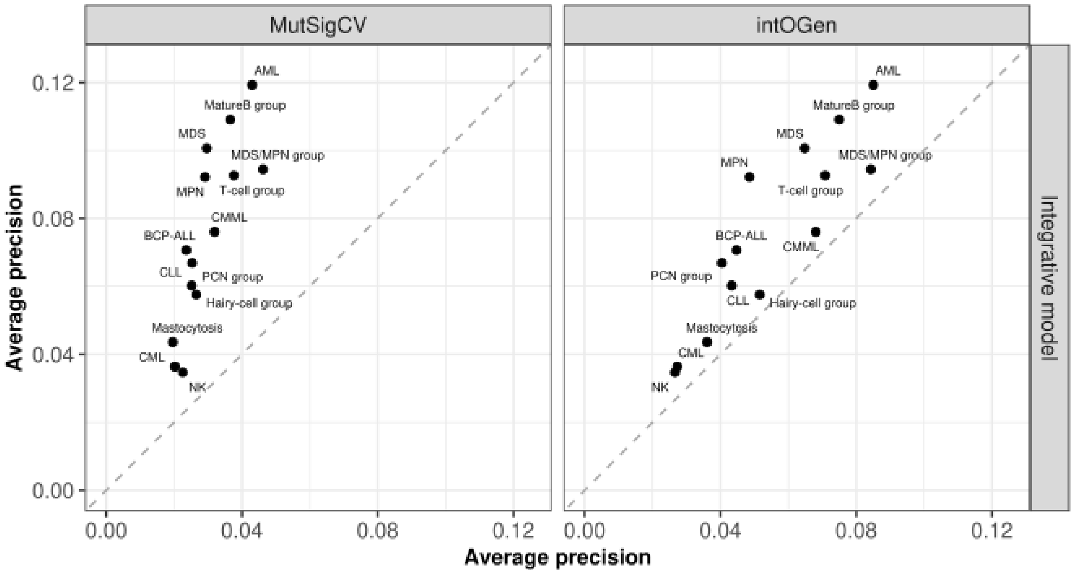

Figure S26. Average precision of the integrative model against MutSigCV and IntOGen among study groups. Across all study groups, our integrative model outperformed IntOGen or MutSigCV.

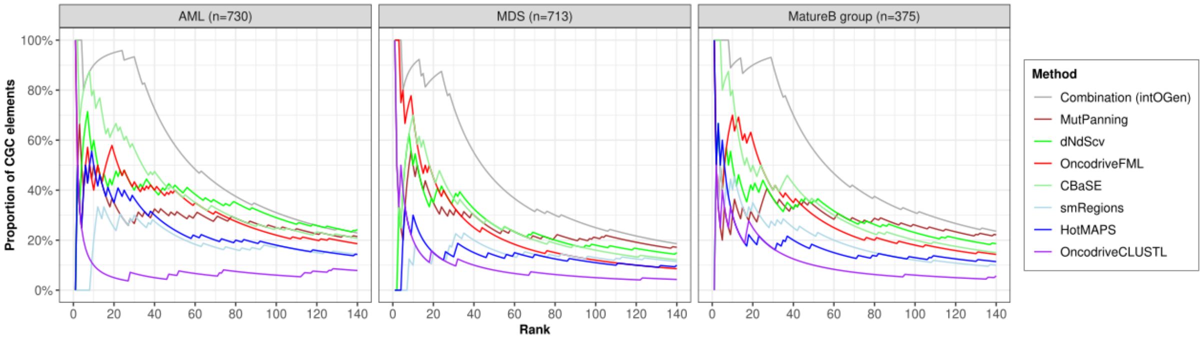

Figure S27. Benchmark of the seven IntOGen tools and their combination using MLL filtered variants. The proportion of CGC drivers among the top-ranking genes in the combined list is greater than that of the lists of individual driver identification methods in three exemplary MLL study groups (AML, MDS, and MatureB group). The proportion of CGC drivers in each list of genes is measured across growing top-ranked genes (x-axis). The MLL variants were filtered using QUALITY=='PASS, discard gnomAD MAF >= 0.0005, keep VAF >= 0.15, and sequencing depth>=20.

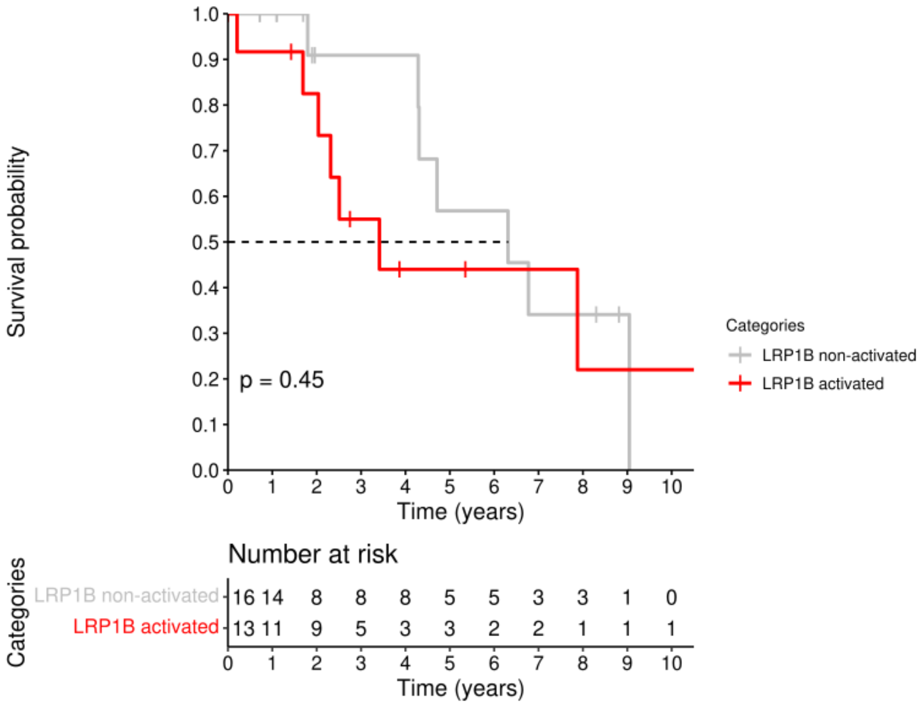

Figure S28. Kaplan-Meier curve estimating the survival between *LRP1B*-activated and non-activated samples within HCL-V. A trend towards shorter overall survival of patients with *LRP1B* activation is observed.

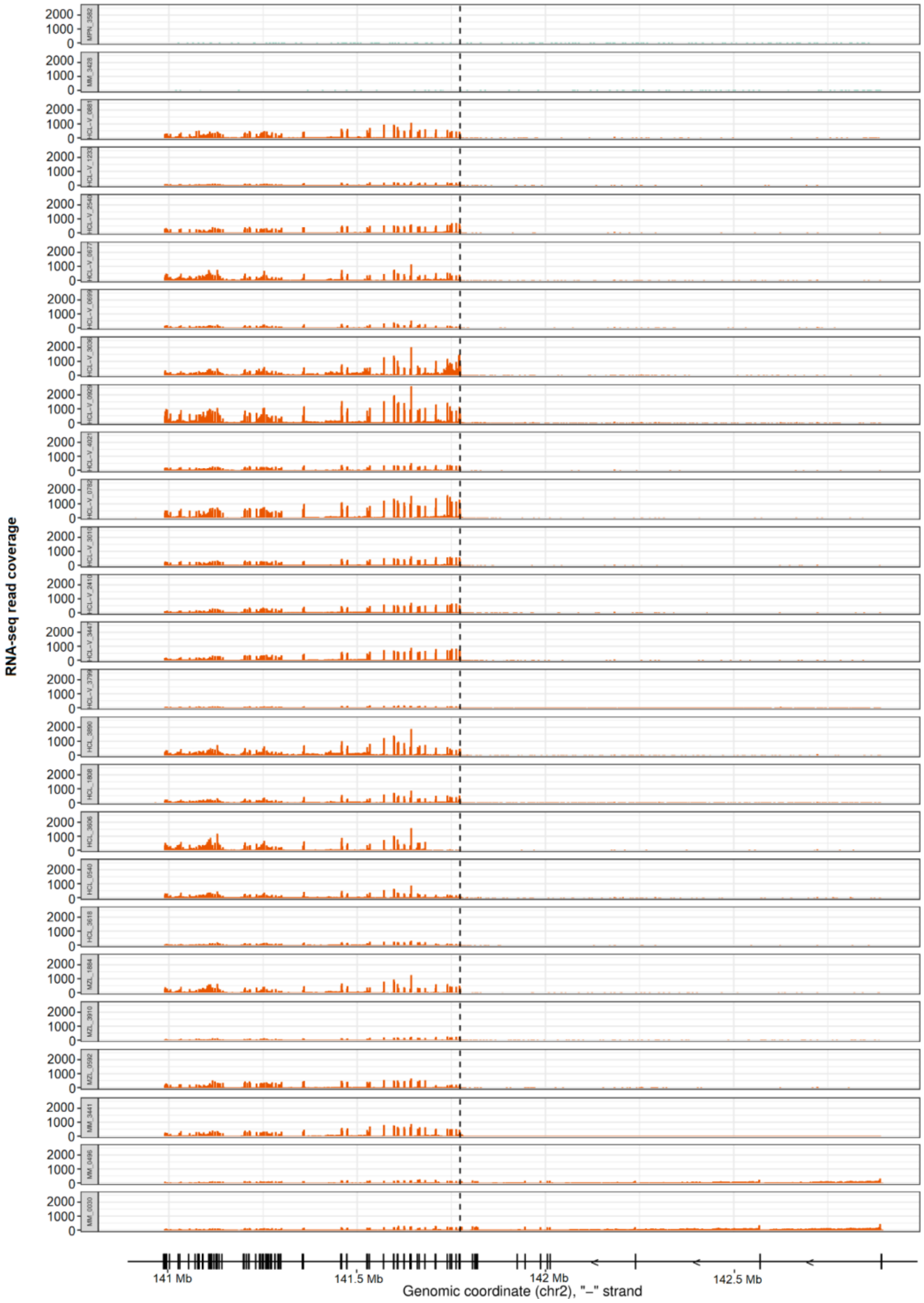

Figure S29. RNA-seq read coverage of *LRP1B* in all *LRP1B*-activated samples (orange) and two *LRP1B*-non-activated examples (green, top 2 rows) in the dataset. Gene annotation of *LRP1B* (GRCh37) is shown at the bottom.

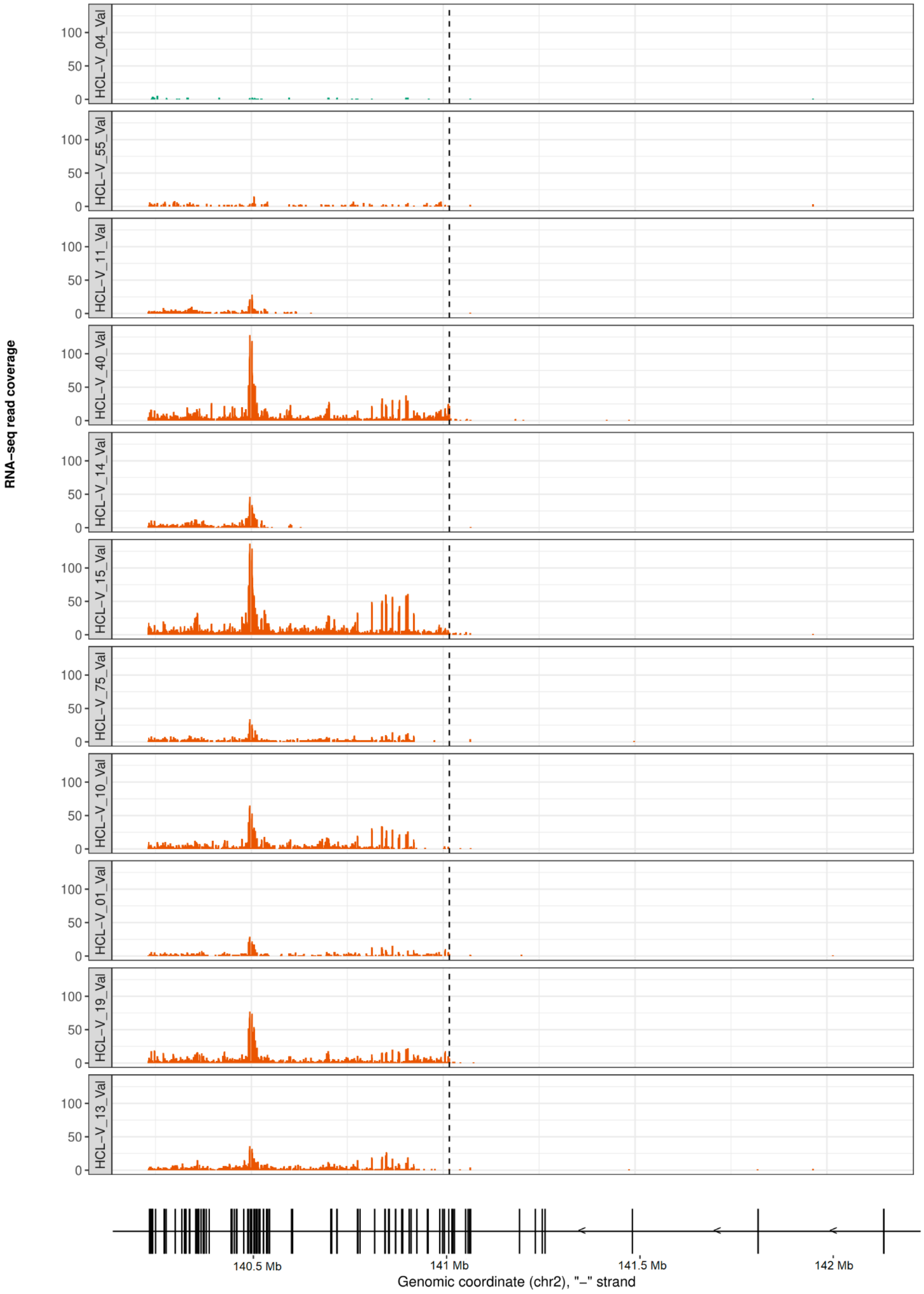

Figure S30. RNA-seq read coverage of *LRP1B* in all *LRP1B*-activated samples (orange) and one *LRP1B*-non-activated example (green, top row) in the validation dataset. Gene annotation of *LRP1B* (GRCh38) is shown at the bottom.

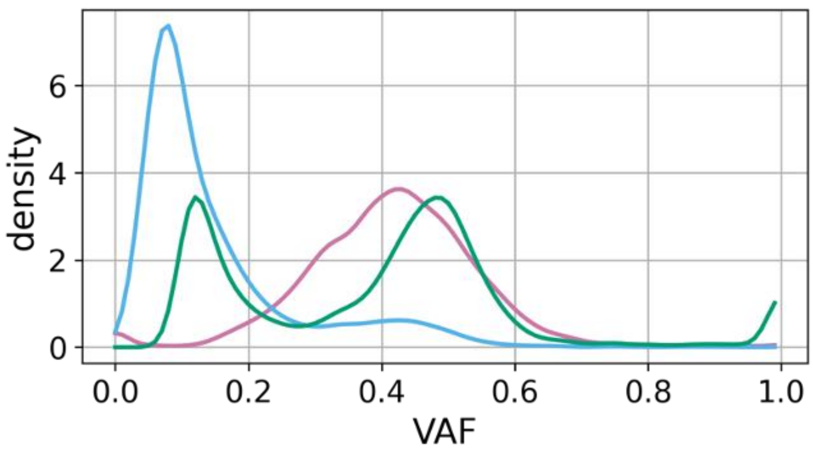

Figure S31. Kernel density plot showing the distribution of VAF. Purple: TCGA-LAML (n=43), WGS, matched normal. Blue: MLL (n=57, 48 AML, 5 CML, 3 CLL, and 1 MCL), WGS, matched normal after filtering for QUALITY==’PASS’. Green: MLL-AML (n=730), WGS, unmatched normal after filtering for QUALITY==’PASS’; discard MAF >= 0.000,5; keep VAF >= 0.10.

References

1. Raczy C, Petrovski R, Saunders CT, Chorny I, Kruglyak S, Margulies EH, et al. Isaac: ultra-fast whole-genome secondary analysis on Illumina sequencing platforms. Bioinformatics. 2013 Aug 15;29(16):2041–3.
2. Kim S, Scheffler K, Halpern AL, Bekritsky MA, Noh E, Källberg M, et al. Strelka2: fast and accurate calling of germline and somatic variants. Nat Methods. 2018 Aug;15(8):591–4.
3. Chen X, Schulz-Trieglaff O, Shaw R, Barnes B, Schlesinger F, Källberg M, et al. Manta: rapid detection of structural variants and indels for germline and cancer sequencing applications. Bioinformatics. 2016 Apr 15;32(8):1220–2.
4. Genomics in the Cloud [Book] [Internet]. [cited 2023 May 16]. Available from: https://www.oreilly.com/library/view/genomics-in-the/9781491975183/
5. Dobin A, Davis CA, Schlesinger F, Drenkow J, Zaleski C, Jha S, et al. STAR: ultrafast universal RNA-seq aligner. Bioinformatics. 2013 Jan 1;29(1):15–21.
6. Uhrig S, Ellermann J, Walther T, Burkhardt P, Fröhlich M, Hutter B, et al. Accurate and efficient detection of gene fusions from RNA sequencing data. Genome Res. 2021 Mar 1;31(3):448–60.
7. Haas BJ, Dobin A, Li B, Stransky N, Pochet N, Regev A. Accuracy assessment of fusion transcript detection via read-mapping and de novo fusion transcript assembly-based methods. Genome Biol. 2019 Oct 21;20(1):213.
8. Anders S, Huber W. Differential expression analysis for sequence count data. Nat Preced. 2010 Mar 15;1–1.
9. Sondka Z, Bamford S, Cole CG, Ward SA, Dunham I, Forbes SA. The COSMIC Cancer Gene Census: describing genetic dysfunction across all human cancers. Nat Rev Cancer. 2018 Nov;18(11):696–705.
10. Alaggio R, Amador C, Anagnostopoulos I, Attygalle AD, Araujo IB de O, Berti E, et al. The

5th edition of the World Health Organization Classification of Haematolymphoid Tumours: Lymphoid Neoplasms. Leukemia. 2022 Jul;36(7):1720–48.

11. Khoury JD, Solary E, Abla O, Akkari Y, Alaggio R, Apperley JF, et al. The 5th edition of the World Health Organization Classification of Haematolymphoid Tumours: Myeloid and Histiocytic/Dendritic Neoplasms. Leukemia. 2022 Jul;36(7):1703–19.
12. Tokheim C, Bhattacharya R, Niknafs N, Gygax DM, Kim R, Ryan M, et al. Exome-Scale Discovery of Hotspot Mutation Regions in Human Cancer Using 3D Protein Structure. Cancer Res. 2016 Jul 1;76(13):3719–31.
13. Arnedo-Pac C, Mularoni L, Muiños F, Gonzalez-Perez A, Lopez-Bigas N. OncodriveCLUSTL: a sequence-based clustering method to identify cancer drivers. Bioinformatics. 2019 Nov 1;35(22):4788–90.
14. Martínez-Jiménez F, Muiños F, López-Arribillaga E, Lopez-Bigas N, Gonzalez-Perez A. Systematic analysis of alterations in the ubiquitin proteolysis system reveals its contribution to driver mutations in cancer. Nat Cancer. 2020 Jan;1(1):122–35.
15. Mularoni L, Sabarinathan R, Deu-Pons J, Gonzalez-Perez A, López-Bigas N. OncodriveFML: a general framework to identify coding and non-coding regions with cancer driver mutations. Genome Biol. 2016 Jun 16;17(1):128.
16. Dietlein F, Weghorn D, Taylor-Weiner A, Richters A, Reardon B, Liu D, et al. Identification of cancer driver genes based on nucleotide context. Nat Genet. 2020 Feb;52(2):208–18.
17. Martincorena I, Raine KM, Gerstung M, Dawson KJ, Haase K, Van Loo P, et al. Universal Patterns of Selection in Cancer and Somatic Tissues. Cell. 2017 Nov 16;171(5):1029-1041.e21.
18. Weghorn D, Sunyaev S. Bayesian inference of negative and positive selection in human cancers. Nat Genet. 2017 Dec;49(12):1785–8.
19. Wainberg M, Kamber RA, Balsubramani A, Meyers RM, Sinnott-Armstrong N, Hornburg D, et al. A genome-wide atlas of co-essential modules assigns function to uncharacterized

genes. Nat Genet. 2021 May;53(5):638–49.

20. Brechtmann F, Bechtler T, Londhe S, Mertes C, Gagneur J. Evaluation of input data modality choices on functional gene embeddings. NAR Genomics Bioinforma. 2023 Dec;5(4):lqad095.
21. Pedregosa F, Varoquaux G, Gramfort A, Michel V, Thirion B, Grisel O, et al. Scikit-learn: Machine Learning in Python. J Mach Learn Res. 2011;12(85):2825–30.
22. Chen T, Guestrin C. XGBoost: A Scalable Tree Boosting System. In: Proceedings of the 22nd ACM SIGKDD International Conference on Knowledge Discovery and Data Mining [Internet]. New York, NY, USA: Association for Computing Machinery; 2016 [cited 2024 Feb 9]. p. 785–94. (KDD '16). Available from: https://dl.acm.org/doi/10.1145/2939672.2939785
23. Virtanen P, Gommers R, Oliphant TE, Haberland M, Reddy T, Cournapeau D, et al. SciPy 1.0: fundamental algorithms for scientific computing in Python. Nat Methods. 2020 Mar;17(3):261–72.
24. Paszke A, Gross S, Massa F, Lerer A, Bradbury J, Chanan G, et al. PyTorch: An Imperative Style, High-Performance Deep Learning Library. In: Advances in Neural Information Processing Systems [Internet]. Curran Associates, Inc.; 2019 [cited 2024 Feb 9]. Available from: https://proceedings.neurips.cc/paper_files/paper/2019/hash/bdbca288fee7f92f2bfa9f7012727740-Abstract.html
25. Cancer Genome Atlas Research Network, Ley TJ, Miller C, Ding L, Raphael BJ, Mungall AJ, et al. Genomic and epigenomic landscapes of adult de novo acute myeloid leukemia. N Engl J Med. 2013 May 30;368(22):2059–74.
26. Costello M, Pugh TJ, Fennell TJ, Stewart C, Lichtenstein L, Meldrim JC, et al. Discovery and characterization of artifactual mutations in deep coverage targeted capture sequencing data due to oxidative DNA damage during sample preparation. Nucleic Acids Res. 2013 Apr 1;41(6):e67.
